# Unravelling the effects of tropical land use conversion on the soil microbiome

**DOI:** 10.1186/s40793-020-0353-3

**Published:** 2020-02-03

**Authors:** Dirk Berkelmann, Dominik Schneider, Anja Meryandini, Rolf Daniel

**Affiliations:** 1 Genomic and Applied Microbiology and Göttingen Genomics Laboratory, Institute of Microbiology and Genetics, Georg-August-University, Grisebachstr. 8, 37077 Göttingen, Germany; 2 Department of Biology, Faculty of Mathematics and Natural Sciences IPB, Bogor Agricultural University, Bogor, Indonesia

**Keywords:** Metagenomics, Oil palm, Soil bacterial communities, Rainforest conversion, Rubber, Soil microbial community

## Abstract

**Background:**

The consequences of deforestation and agricultural treatments are complex and affect all trophic levels. Changes of microbial community structure and composition associated with rainforest conversion to managed systems such as rubber and oil palm plantations have been shown by 16S rRNA gene analysis previously, but functional profile shifts have been rarely addressed. In this study, we analysed the effects of rainforest conversion to different converted land use systems, including agroforestry (“jungle rubber”) and monoculture plantations comprising rubber and oil palm, on soilborne microbial communities by metagenomic shotgun sequencing in Sumatra, Indonesia.

**Results:**

The diversity of bacteria and archaea decreased whereas diversity of fungi increased in the converted land use systems. The soil microbiome was dominated by bacteria followed by fungi. We detected negative effects of land use conversion on the abundance of *Proteobacteria* (especially on *Rhizobiales* and *Burkholderiales*) and positive effects on the abundance of *Acidobacteria* and *Actinobacteria*. These abundance changes were mainly driven by pH, C:N ratio, and Fe, C and N content. With increasing land use intensity, the functional diversity decreased for bacteria, archaea and fungi. Gene abundances of specific metabolisms such as nitrogen metabolism and carbon fixation were affected by land use management practices. The abundance of genes related to denitrification and nitrogen fixation increased in plantations while abundance of genes involved in nitrification and methane oxidation showed no significant difference. Linking taxonomic and functional assignment per read indicated that nitrogen metabolism-related genes were mostly assigned to members of the *Rhizobiales* and *Burkholderiales*. Abundances of carbon fixation genes increased also with increasing land use intensity. Motility- and interaction-related genes, especially genes involved in flagellar assembly and chemotaxis genes, decreased towards managed land use systems. This indicated a shift in mobility and interspecific interactions in bacterial communities within these soils.

**Conclusions:**

Rainforest conversion to managed land use systems drastically affects structure and functional potential of soil microbial communities. The decrease in motility- and interaction-related functions from rainforest to converted land use systems indicated not only a shift in nutrient cycling but also in community dynamics. Fertilizer application and correspondingly higher availability of nutrients in intensively managed plantations lead to an environment in which interspecific interactions are not favoured compared to rainforest soils. We could directly link effects of land management, microbial community structure and functional potential for several metabolic processes. As our study is the first study of this size and detail on soil microbial communities in tropical systems, we provide a basis for further analyses.

## Background

Conversion of natural systems to agriculturally managed land use systems is constantly increasing worldwide [1, 2]. Indonesia is one of the world’s largest palm oil and rubber producer and harbours a high biodiversity in tropical rainforests. Thus, effects of land use changes and rainforest conversion to agriculturally managed systems on biodiversity and ecosystem functions are of high interest including conflicts and trade-offs between conservation of biodiversity and economic revenue. In recent years, studies targeting different trophic levels as well as biogeochemical, ecological and socioeconomical effects of the conversion have been published [3, 4]. Soil microbial communities are integral components of terrestrial ecosystems. Microorganisms in soils comprise prokaryotes (archaea and bacteria), fungi and protists. Ecosystem functioning depends to a large extent on the functional diversity and activity of the belowground microbial system [5, 6]. In addition, microorganisms play a key role in decomposing soil organic matter and mineralizing nutrients in soil [7]. It has been shown that rainforest conversion to plantations has negative effects on the biodiversity of fungi [8, 9], protists [10], vertebrates [11], insects and plants [12–14], archaea [15], but not on bacterial diversity which increased [15, 16]. Additionally, the microbial community composition was severely affected by these conversion processes with a high impact on *Proteobacteria* which showed an abundance decrease and *Actinobacteria,* which showed an abundance increase with increasing land use intensity [15, 16]. Furthermore, negative effects on aboveground and belowground carbon stocks, CO_2_ fluxes and leaching in these converted land use systems were shown before [17–21]. Management practices such as application of fertilizer, liming, application of herbicides (e.g. glyphosate) and harvesting in turn affect functions of microbial communities [22–24].

Previous studies targeting soil microbial communities in the tropics often relied on the analysis of markers like the 16S rRNA gene. This is cost-efficient, taxonomically accurate and relatively fast but functional profiles can only be predicted based on taxonomic information using bioinformatic tools such as Tax4Fun2 [25], Piphillin [26] and PiCrust [15, 27–31]. Prediction-based analysis of microbiome functions provides first insights into environmental microbial processes such as nutrient cycling and emission of climate gases influenced by agricultural management [16]. While these studies provided a first impression of the functional potential, direct sequencing of metagenomic DNA and identification of functional genes is needed to verify prediction-based findings [32]. However, studies covering functional analyses of rainforest conversion to oil palm monocultures based on direct sequencing of metagenomes are rare. To our knowledge, only Tripathi et al. [27] analysed microbial functioning in oil palm soils so far, but with a focus on logging effects. Effects of rainforest conversion to jungle rubber and rubber plantations systems on functional gene profiles, as analysed in this study, were not addressed yet. In a previous study, which was based on taxonomy-derived functional predictions, it was suggested that nutrient cycling related processes such as nitrogen fixation, denitrification and methane oxidation as well as motility- and interaction-related processes such as chemotaxis and type IV secretion systems are negatively affected by rainforest conversion to managed systems. It was hypothesized that fertilizer input and liming in intensively managed systems such as rubber and oil palm plantations reduces the need for nutrient acquisition, and thereby influencing interactions, mobility and communication of soilborne bacterial communities [16]. Even if taxonomic and functional profiles are studied, it is still not known which taxonomic group is responsible for which processes, leaving a gap that needs to be addressed as well.

To gain deeper insights into these processes, we analysed rainforest conversion to jungle rubber, rubber plantations and oil palm plantations by direct sequencing of the corresponding soil metagenomes. Subsequently, taxonomic and functional profiles in the different land use systems were analysed and compared. We linked functional results with their corresponding taxonomic background. Based on our previous studies analysing the 16S rRNA marker gene sequences in the same sampling sites and studies on similar systems in Southeast Asia [9, 10, 15, 16, 18, 33], we formulated three hypotheses. We assume that diversity will decrease for soilborne archaea, Eukaryotes and increase for bacteria with increasing land use intensity (H1). Furthermore, we expect functional profiles to differ between rainforest and plantation monocultures, in which nitrogen and methane metabolism as well as motility- and interaction-related processes are negatively affected by the reduced aboveground diversity in plantations (H2). Furthermore, we hypothesize that strongly affected taxonomic groups like *Proteobacteria* and *Actinobacteria* are linked to crucial processes in nutrient cycling such as nitrogen-fixation, nitrification and methane oxidation. In addition, some of these affected groups are indicators for motility-related processes like chemotaxis and quorum sensing (H3).

## Results

### General characteristics of the soil metagenome dataset

**Fig. 1:**
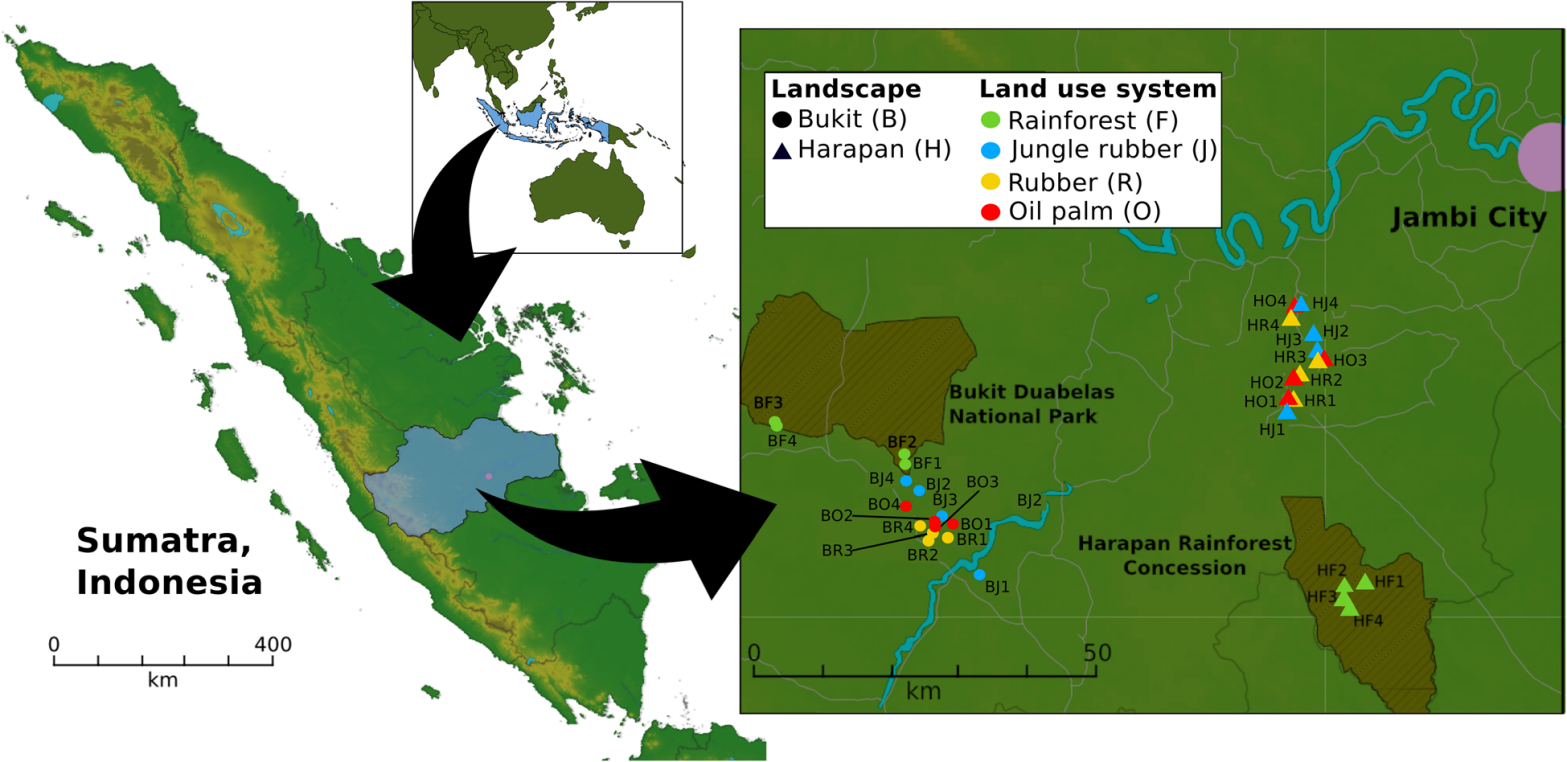
Sampling sites in the province of Jambi on Sumatra, Indonesia. Four core plots with three subplots per core plot in each converted land use system and rainforest reference sites were studied. The core plot design was established in two landscapes within the province of Jambi. The landscapes Bukit Duabelas and Harapan are indicated by “B” and “H” in the Plot ID with “F” for rainforest, “J” for jungle rubber, “R”, rubber and “O” for oil palm

Metagenome sequencing and quality-filtering of the 32 Indonesian soil samples covering the land use types rainforest, jungle rubber, rubber plantation and oil palm plantation in two landscapes (see Methods for details, Fig. 1) resulted in more than 1.11 billion high-quality reads in total and approximately 33 million reads on average per sample (Additional file 1: Table S1). The average read length per paired-end read was 146 bp with an average GC content of 60%. To get an overview of the taxonomic community structure, reads were first clustered at domain level (Additional file 2: Figure S1). Approximately 40% of all reads could not be taxonomically classified. We recorded a bacterial dominance in bulk soils of all land use systems including the rainforest controls with abundances ranging from 48.5 to 52.5% of all reads (524,556,933 reads in total). Highest bacterial abundances were detected in rainforest samples (51% in Bukit and 52.5% in Harapan), which also contained the lowest number of unclassified reads of all sample types. Approximately 7% of all sequences were classified as *Eukaryota* (78,682,140 reads in total), while less than 0.6% of all reads were classified as archaea (4,251,297 reads in total) and viruses (1,147,105 reads).

### Effects of rainforest conversion on microbial diversity

**Fig. 2:**
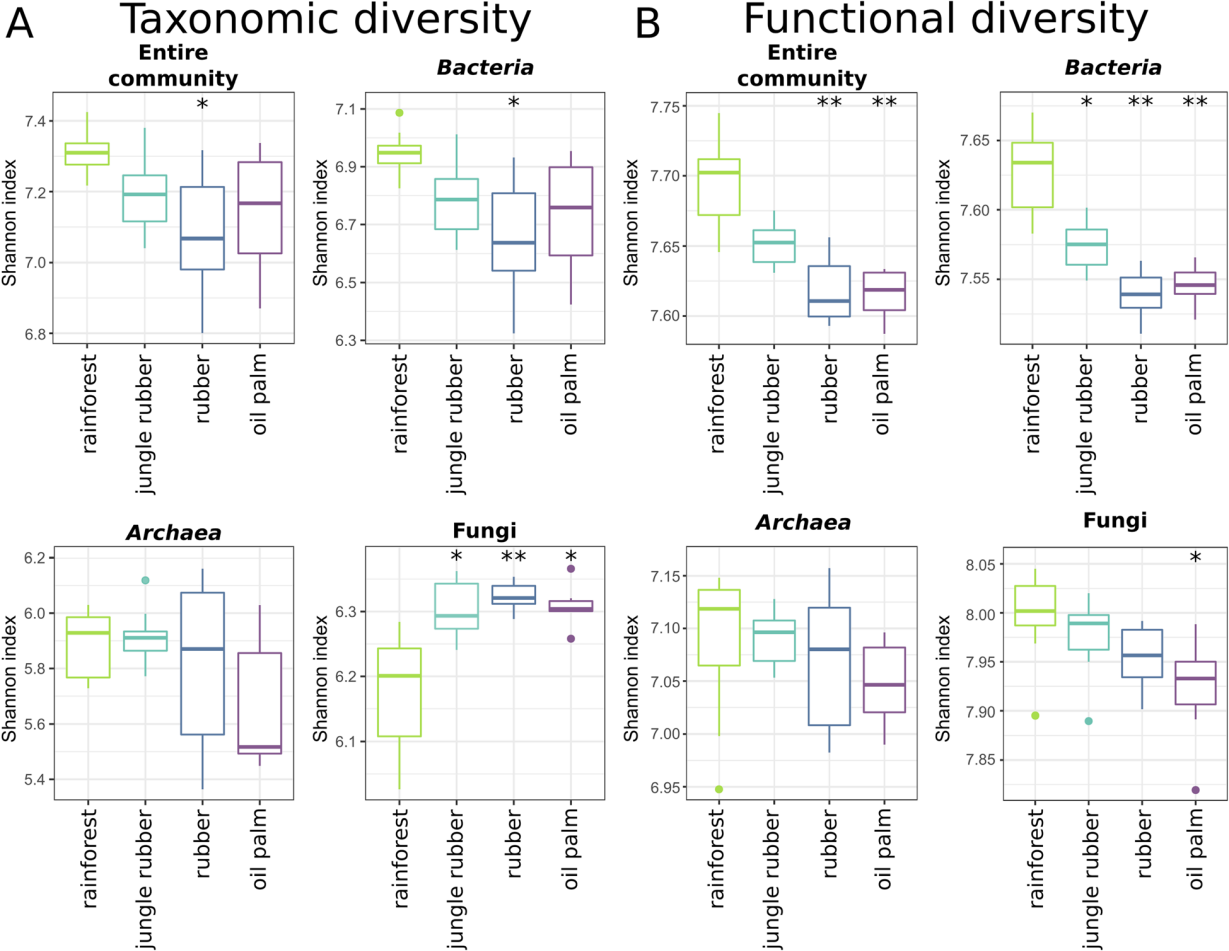
Taxonomic (**a**) and functional diversity (**b**) of the microbial communities in the land use systems. Shannon diversity indices were calculated for the entire community as well as bacteria, archaea and fungi in each land use system. Significant changes in each land use system compared to rainforest are indicated with * (* = *p* < 0.05, ** = *p* < 0.001, *** = *p* < 0.0001)

We analysed taxonomic and functional diversity of the entire soil community and separately of bacteria, archaea and fungi and compared the results derived from all converted land use systems with those of rainforest by using the Shannon index for each land use type (Fig. 2). Taxonomic diversity decreased for all prokaryotes from rainforest to the monoculture land uses rubber and oil palm (Fig. 2a). Lowest values for the entire community and bacteria, and archaea were detected in rubber and oil palm soils, respectively (with *p* < 0.05 compared to rainforest). In contrast, fungi showed an opposite trend with an increase in diversity from rainforest to converted systems with the highest diversity in rubber (all p < 0.05). We additionally analysed functional diversity on gene level (Fig. 2b). We observed gradual decreases in functional diversity from rainforest over jungle rubber to plantations for all analysed groups. As recorded for taxonomic diversity of the entire community and bacteria, lowest values for functional diversity were obtained in rubber. For both groups, the detected decreases from rainforest to both monoculture systems were significant with *p* < 0.001. Although functional diversity decreased for archaea as well, changes were less pronounced and with high variance in rubber. Contrary to taxonomic diversity, functional diversity for fungi decreased from rainforest to converted systems with lowest values in oil palm samples (*p* < 0.05).

### Effects of rainforest conversion on microbial community structures

Studying the microbial community composition at high taxonomic resolution based solely on short metagenomic reads is challenging. In order to provide a clear but still detailed analysis and avoid overinterpretation at species level, we chose order level to compare the communities. Differential abundance analysis of the soil bacterial community composition showed that significant changes between the different land use systems were more pronounced in the Harapan landscape than in the Bukit landscape (Additional file 3: Figure S2).

**Fig. 3:**
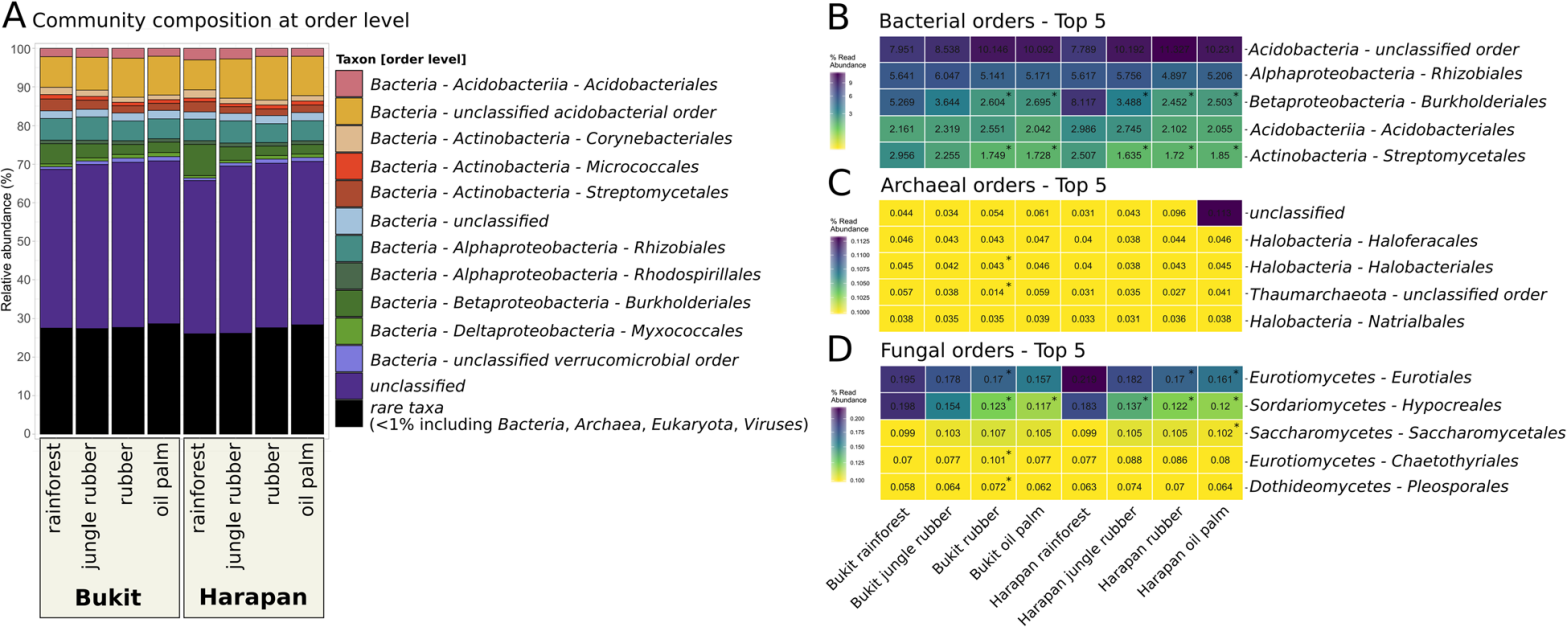
Community composition displayed as relative abundances at order level of the entire community, bacteria, archaea and fungi. (**a**) The entire community including all used sequences is shown in bars in which all orders below 1% were clustered as “rare taxa”. The five most abundant orders within the bacteria (**b**), archaea (**c**) and fungi (**d**) are shown seperately. Significant differences (p < 0.05) compared to rainforest are marked with *

The most abundant bacterial orders were uncultured acidobacterial orders, *Rhizobiales*, *Acidobacteriales*, *Burkholderiales* and *Streptomycetales* (Fig. 3a, b). Abundances of *Rhizobiales* and *Acidobacteriales* were high in general but did not show significant changes in the converted land use systems. *Burkholderiales*, which are involved in denitrification [34] and nitrogen fixation [35], showed the largest differences in abundances between the different land use systems of all bacterial orders. Abundances of *Burkholderiales* decreased significantly in both landscapes from rainforest to the monoculture systems with generally higher abundances in Harapan samples (5.2 to 2.6% in Bukit and 8.1 to 2.5% in Harapan, *p* < 0.05).

The most abundant orders within the archaea belonged to the *Halobacteria* and *Thaumarchaeota*, with the largest fraction belonging to unclassified archaea (Fig. 3c). *Haloferacalaes*, *Halobacteriales* and *Natrialbales* of the *Halobacteria* were detected in all land use systems with similar abundances and without significant changes. In general, significant changes were not detected for archaeal orders in all Harapan samples whereas in Bukit soils 16 of all detected 62 orders differed significantly between rainforest and converted land use systems.

The most abundant fungal orders were *Eurotiales* and *Hypocreales*, which both belong to the *Ascomycota* (Fig. 3d). *Hypocreales* decreased significantly in abundance from rainforest to all converted land use systems, except Bukit jungle rubber. *Hypocreales*decreased from 0.19% in Bukit rainforest and 0.18% in Harapan rainforest to 0.12% in rubber of both landscapes and 0.11 and 0.12% in oil palm plantations, respectively (*p* < 0.05). *Eurotiales* showed significant abundance changes compared to rainforest in rubber and oil palm plantations of both landscapes. Increasing abundances along the land use gradient from rainforest to converted systems were also observed for *Chaetothyriales* and *Pleosporales* with p.adj < 0.05 in Bukit rubber samples.

We compared trophic groups of protists in each land use system (0.09% of all reads) using the same functional categories described by Schulz et al. [10] (Additional file 4: Figure S3). Phagotrophs were the most abundant group (37 to 39%) followed by photoautotrophs (29 to 30%) and animal parasites (27 to 31%). A notable increase of protistan animal parasites compared to rainforest reference sites was recorded in jungle rubber soils, whereas abundances of the phagotrophs and photoautotrophs remained rather stable. In contrast, results by Schulz et al. [10], which were based on 18S rRNA gene amplicons, showed an increase of phagotrophs and a decrease for animal parasites in converted systems compared to rainforest samples.

Additionally, microbiome composition was compared at order level based on extracted rRNA sequences (Additional file 5: Figure S4). Similar community structures were observed as in previous 16S rRNA marker gene studies [15, 16]. Similar to the phylogenetically assigned metagenome data, *Proteobacteria* and *Acidobacteria* were the most abundant bacterial phyla in extracted rRNA sequences. Notably, the fraction of unclassified bacteria at phylum level was higher in extracted rRNA sequences, ranging from 20.2 to 26.5% while remaining under 2.2% in shotgun data. Pairwise PERMANOVA analysis showed no significant differences between shotgun data and rRNA sequence controls.

### Influence of abiotic parameters on soilborne communities

**Fig. 4:**
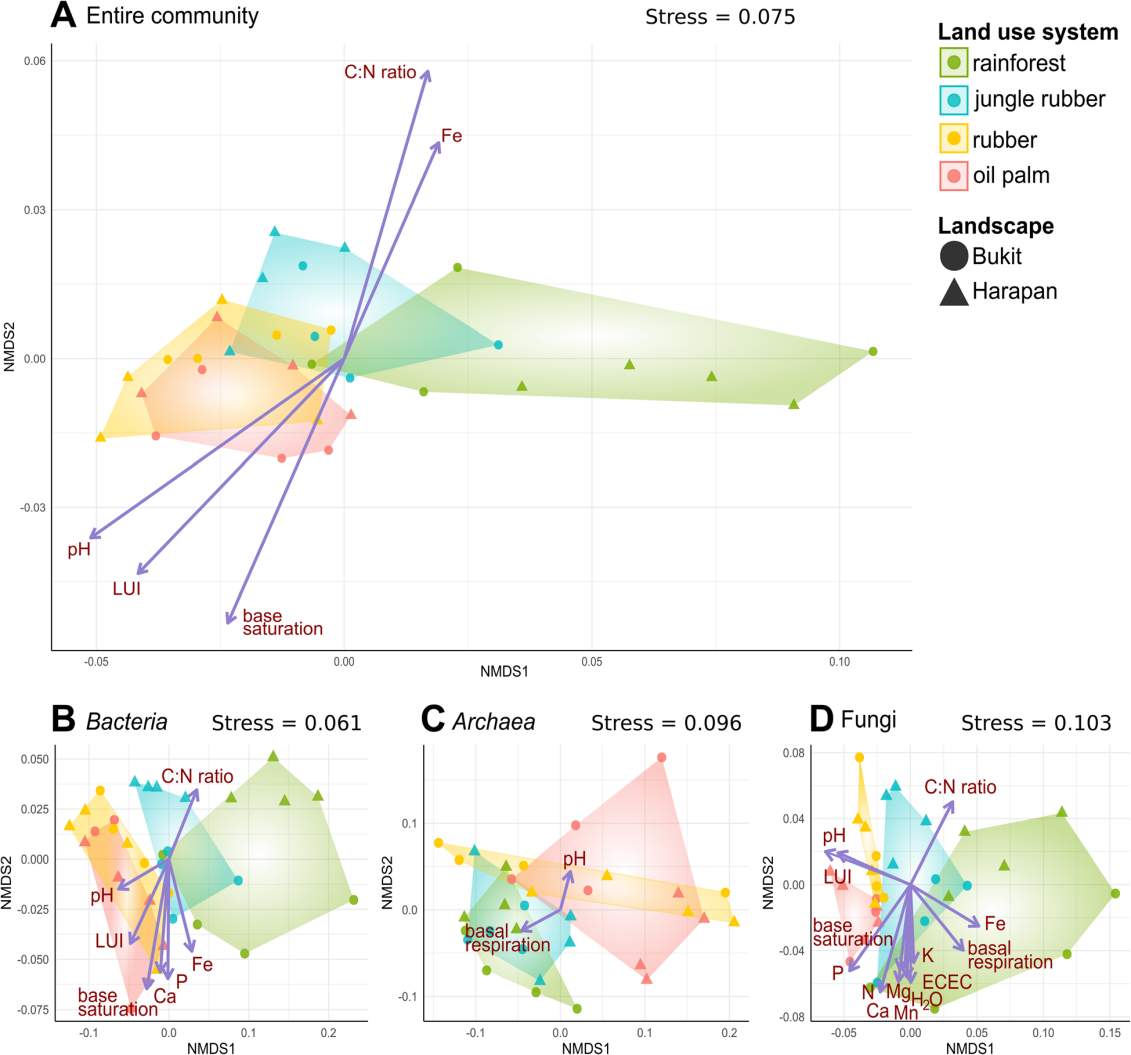
Non-metric multidimensional scaling of the entire (**a**), bacterial (**b**), archaeal (**c**) and fungal (**d**) community in all land use systems. The ordinations are based on Bray Curtis dissimilarity matrices including analysis of abiotic factors by employing an environmental fit. Only abiotic factors with p < 0.05 were included

The composition of microbial communities in soil is tightly connected with soil characteristics and nutrient availability. These parameters are in turn connected with land use and management practices [36–39]. In order to investigate the impact of soil attributes on soilborne communities with respect to rainforest conversion, we employed nonparametric multidimensional scaling (NMDS) on Bray Curtis dissimilarity matrices (Fig. 4).

Multivariate analysis of the entire soil microbial community composition showed a gradient along the four different tested land use systems corresponding to increasing land use intensity (rainforest < jungle rubber < rubber plantation < oil palm plantation). NMDS stress levels ranged from 0.06 to 0.1. The parameters which correlated with the ordination of the entire community were C:N ratio, land use index (LUI), base saturation, iron content and pH (*p* < 0.05) (Additional file 6: Table S2). Similar clustering patterns were obtained for bacteria, archaea and fungi, which are all in line with the proposed land use gradient. Abiotic parameters that showed correlation with the bacterial community were LUI, pH, C:N ratio, Fe, Ca, P and base saturation (for details see, Additional file 6: Table S2). The LUI, pH value, P content and Ca content increased with higher land use intensity, reflecting the effects of agricultural liming and fertilizer application [9, 18]. These factors correlated also with the fungal community composition in addition to nitrogen, Mn, K and Mg content, water saturation, effective cation exchange (ECEC), and basal respiration. The only detected correlations to archaea were pH and basal respiration.

### Impact of rainforest conversion on microbiome functions

**Fig. 5:**
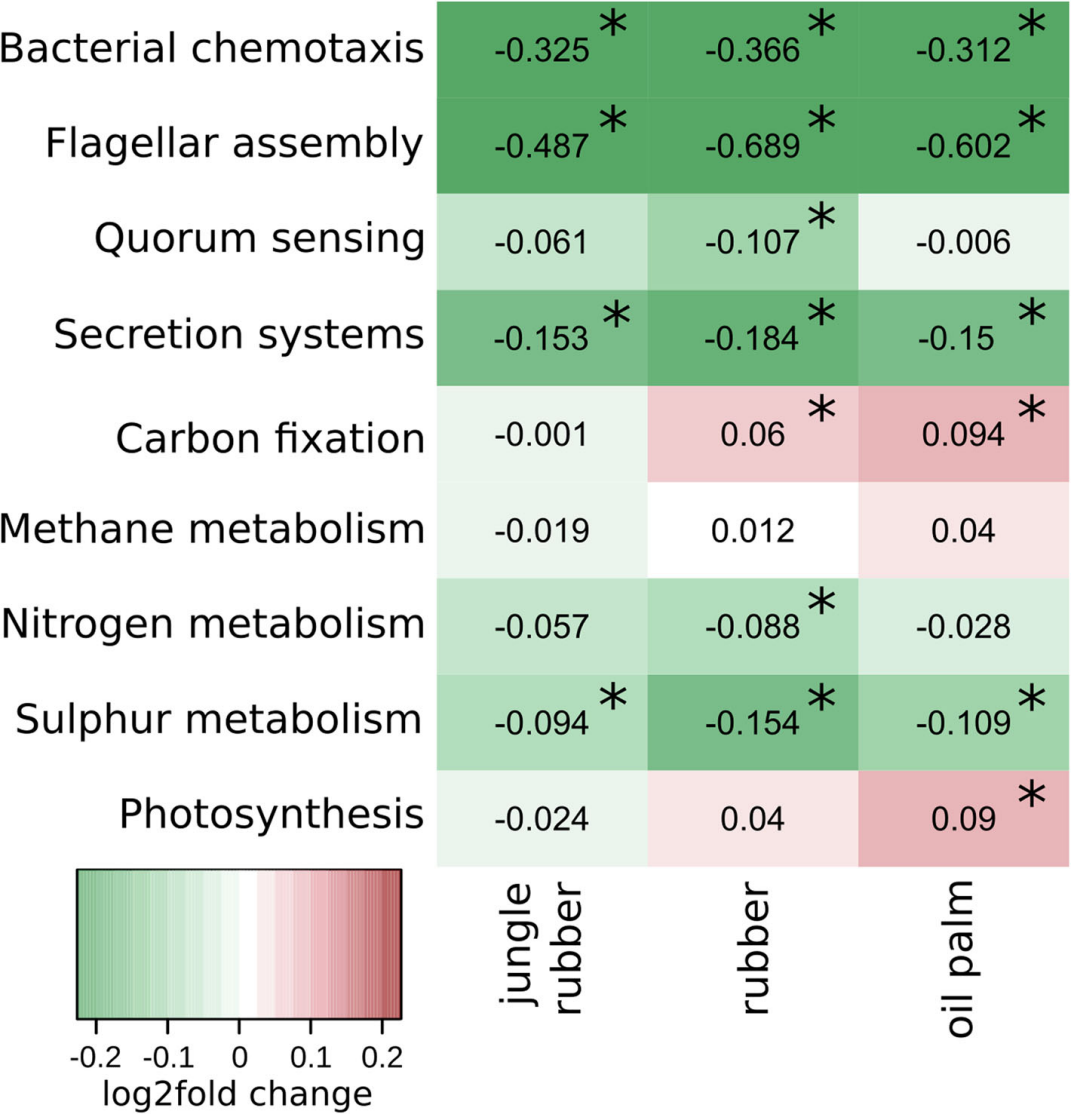
Functional profile of selected metabolisms based on KEGG categories (level 3) displayed as log2fold changes. Negative and positive log2fold changes indicate decreased and increased abundances in the corresponding converted land use systems compared to rainforest samples. Values with p.adj < 0.05 are marked with *

**Fig. 6:**
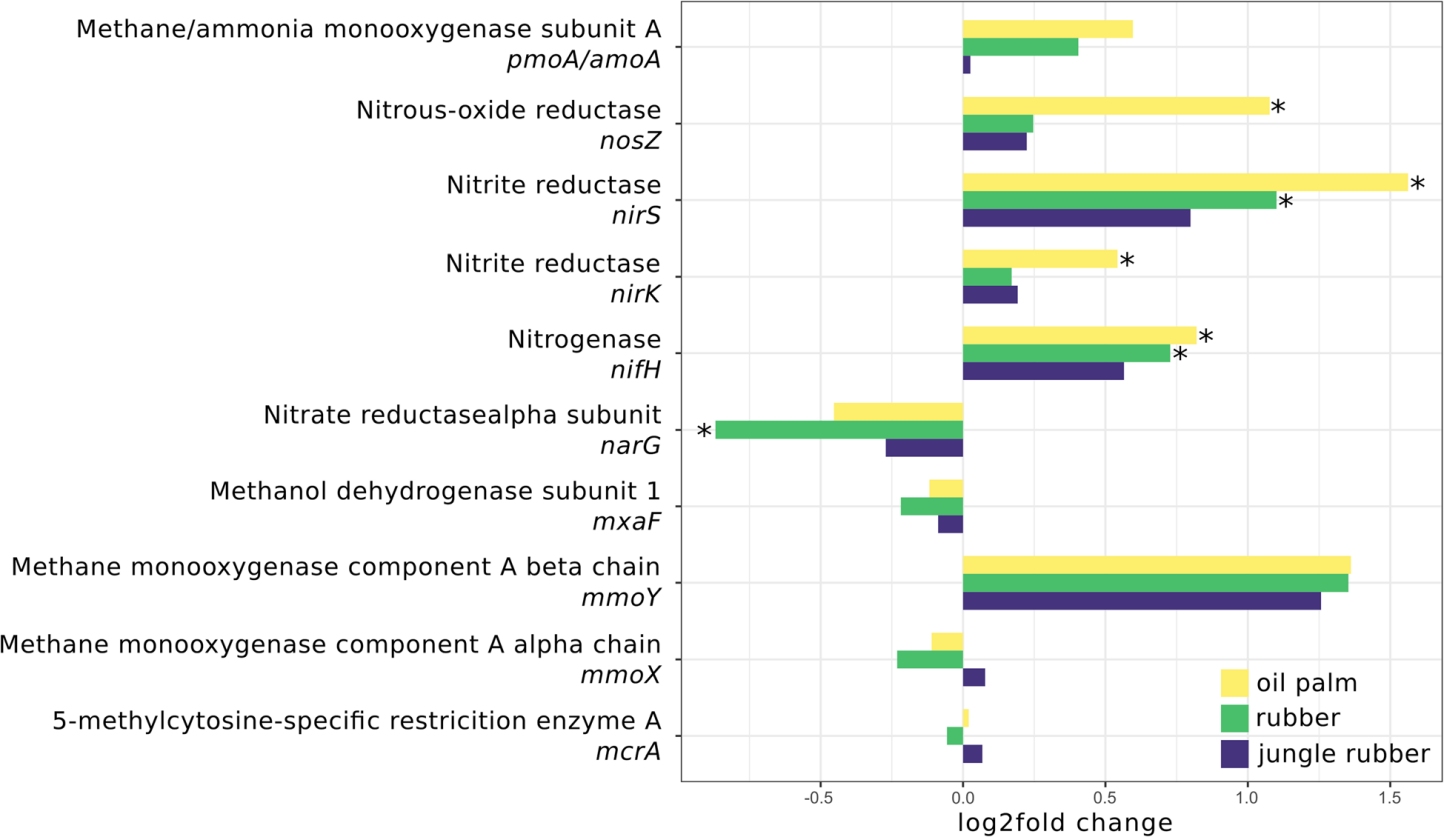
Log2fold changes of selected marker genes of nitrogen and methane metabolism for each analysed land use system compared to rainforest. Significant differences (p.adj. < 0.05) are marked with *. Negative and positive log2fold changes indicate decreased and increased abundances in the corresponding converted land use systems compared to rainforest samples

**Fig. 7:**
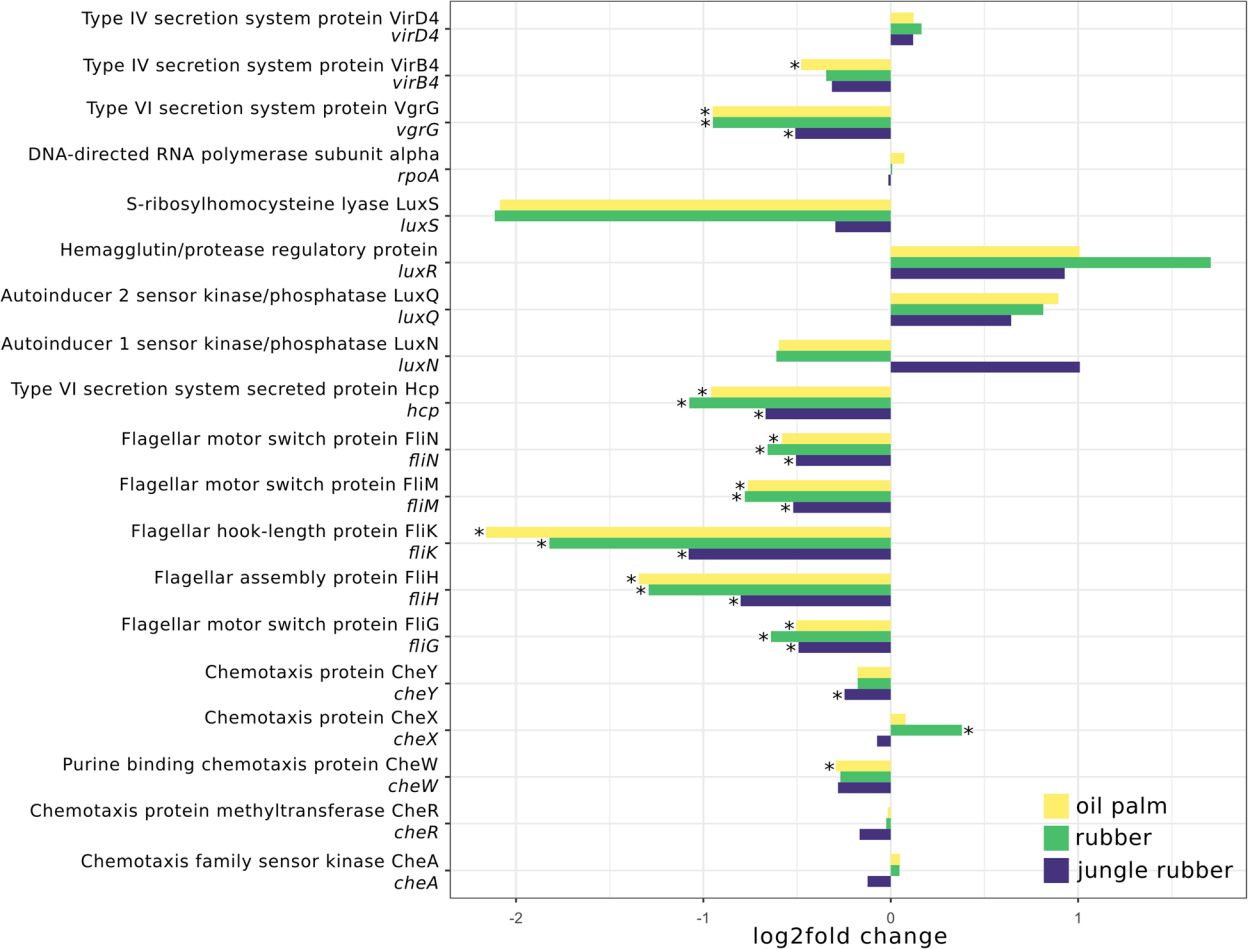
Log2fold changes of selected marker genes of motility related genes for each analysed land use system compared to rainforest. Significant differences (p.adj. < 0.05) are marked with *. Negative and positive log2fold changes indicate decreased and increased abundances in the corresponding converted land use systems compared to rainforest samples

To assess potential functional responses related to the conversion of rainforest, we used differential gene abundance analysis. Significant differences for 7294 genes in all land use systems compared to rainforest were detected. Notably, these detected changes were stronger (positive as well as negative) in Harapan soils compared to Bukit soils (Additional file 7: Figure S5). In fact, changes in Bukit jungle rubber compared to Bukit rainforest were minor compared to all other land uses with only 7 significantly changed genes. One of these was the *nifD* gene, which is involved in nitrogen fixation and encodes a subunit of the nitrogenase enzyme. Similar to the NMDS analysis based on microbial taxonomy, the functional profile differed between rainforest and converted land use systems (Additional file 8: Figure S6). We also analysed functional groups (at level three of the KEGG pathway hierarchy) in each converted land use system compared to rainforest as well as single genes (Figs. 5, 6, 7). The majority of functional differences were detected in Harapan soils. We detected 197 significant abundance changes of functional groups for jungle rubber, 221 for rubber plantation and 108 for oil palm plantation in Harapan soils. In Bukit soils, we detected 177 significant abundance differences of functional groups for rubber and 73 for oil palm but none for jungle rubber. Functional groups covering genes involved in nutrient cycling were analysed in detail including nitrogen metabolism, methane metabolism, carbon fixation and sulphur metabolism (Fig. 5). In addition, functional groups associated with interaction and competition such as chemotaxis, flagellar assembly, quorum sensing, secretion systems and photosynthesis were evaluated.

Nitrogen metabolism-related genes decreased significantly from rainforest to rubber samples with a log2fold change of − 0.08 (*p* < 0.01). Carbon fixation increased from rainforest to rubber (log2fold change 0.06 with *p* < 0.05) and oil palm samples (log2fold change 0.09 with p < 0.01). Sulphur metabolism related genes decreased from rainforest to all converted land use systems, with log2fold changes ranging from − 0.09 in jungle rubber to − 0.1 in oil palm and − 0.15 in rubber. Genes involved in photosynthesis increased from rainforest to oil palm soils with a log2fold change of 0.09 (p < 0.01). At landscape level sulphur metabolism did not change in Bukit jungle rubber and oil palm. Additionally, we did not detect changes for nitrogen metabolism related genes in Harapan rubber samples (Additional file 9: Figure S7).

In order to analyse the potential for interactions, we selected bacterial genes involved in chemotaxis and flagellar assembly for motility, quorum sensing and secretion systems for interactions (Fig. 5 and Additional file 9: Figure S7). Bacterial chemotaxis, flagellar assembly and secretion systems showed decreases in gene abundances from rainforest to all converted land use systems. For quorum sensing, we detected a decrease in abundance only in rubber samples.

### Analysis of specific gene abundances for energy metabolism and motility

Previously analysed pathways were further investigated based on abundances of characteristic genes. Selected genes were divided into two categories related to energy metabolism or motility (Figs. 6, 7 and Additional file 10: Figure S8 and Additional file 11: Figure S9). The denitrification related marker genes *nirK*, *nirS* and *nosZ* increased significantly from rainforest to oil palm plantation, with log2fold changes ranging from 0.5 (*nirK*) to 1.5 (*nirS*). Nitrogen fixation marker *nifH* increased from rainforest to monoculture land use systems (0.7 in rubber and 0.8 in oil palm, *p* < 0.05) and the dissimilatory nitrate reduction marker gene *narG* increased from rainforest to rubber plantations. Other marker genes related to nitrogen or methane metabolism showed no differences compared to rainforest, indicating a higher potential for denitrification and nitrogen fixation in converted land use systems. Since our results suggested that *Rhizobiales* and *Burkholderiales* are of particular importance in the studied rainforest soils due to their connection to nitrogen cycling, the question arose whether this is connected to symbiotic root nodule formation or rather to an endophytic lifestyle. We therefore analysed the abundances of *nod* genes, which are encoding nodulation factors and are crucial for the formation of root nodule symbiosis. However, evidence that rainforest conversion affects abundance of these genes was not found (Additional file 12: Figure S10).

We did not see changes accompanying rainforest conversion for methane metabolism-related genes as a group. Consequently, we analysed marker genes for methane oxidation as well (Fig. 6) but did not recorded significant changes in abundance for all analysed genes.

The abundance of flagellar assembly markers decreased significantly from rainforest to all converted systems (Fig. 7). Similar results were observed for the type VI secretion system proteins *vgrG* and *hcp*, which decreased from rainforest to all converted systems. However, when analysing the landscapes separately, most significant differences were observed in Harapan samples (Additional file 11, Figure S9). Chemotaxis markers did not show such a clear pattern. Chemotaxis gene *cheY* decreased in jungle rubber samples and *cheW* decreased from rainforest to oil palm. In contrast, *cheX* increased from rainforest to rubber samples. Type IV secretion system gene *virB4* only showed significant changes in oil palm with a minor decrease, whereas the quorum sensing marker genes showed no significant changes in abundance compared to rainforest.

### Connecting taxonomy and functions – who does what?

**Fig. 8:**
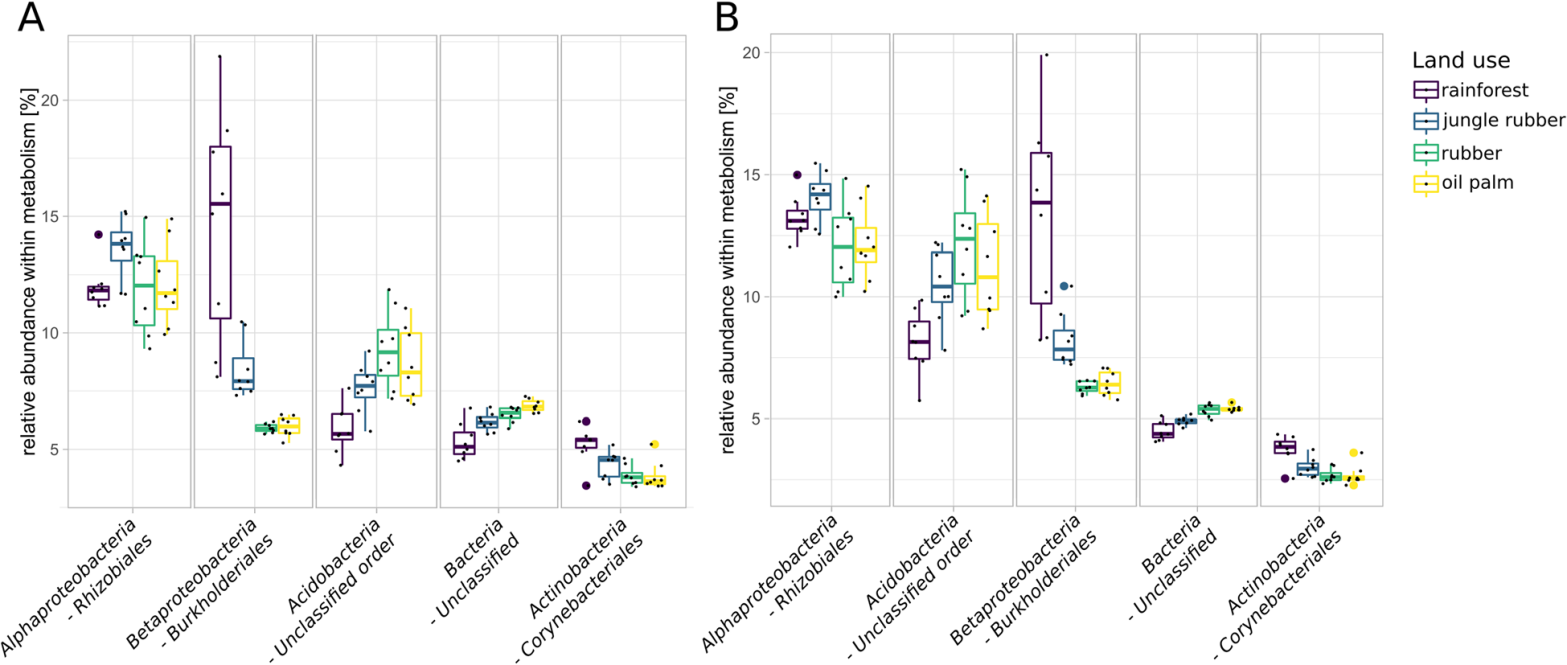
Relative abundances of the top five detected taxonomic orders within nitrogen (**a**) and methane metabolism (**b**) KEGG level 3 categories in each land use system are displayed

**Fig. 9:**
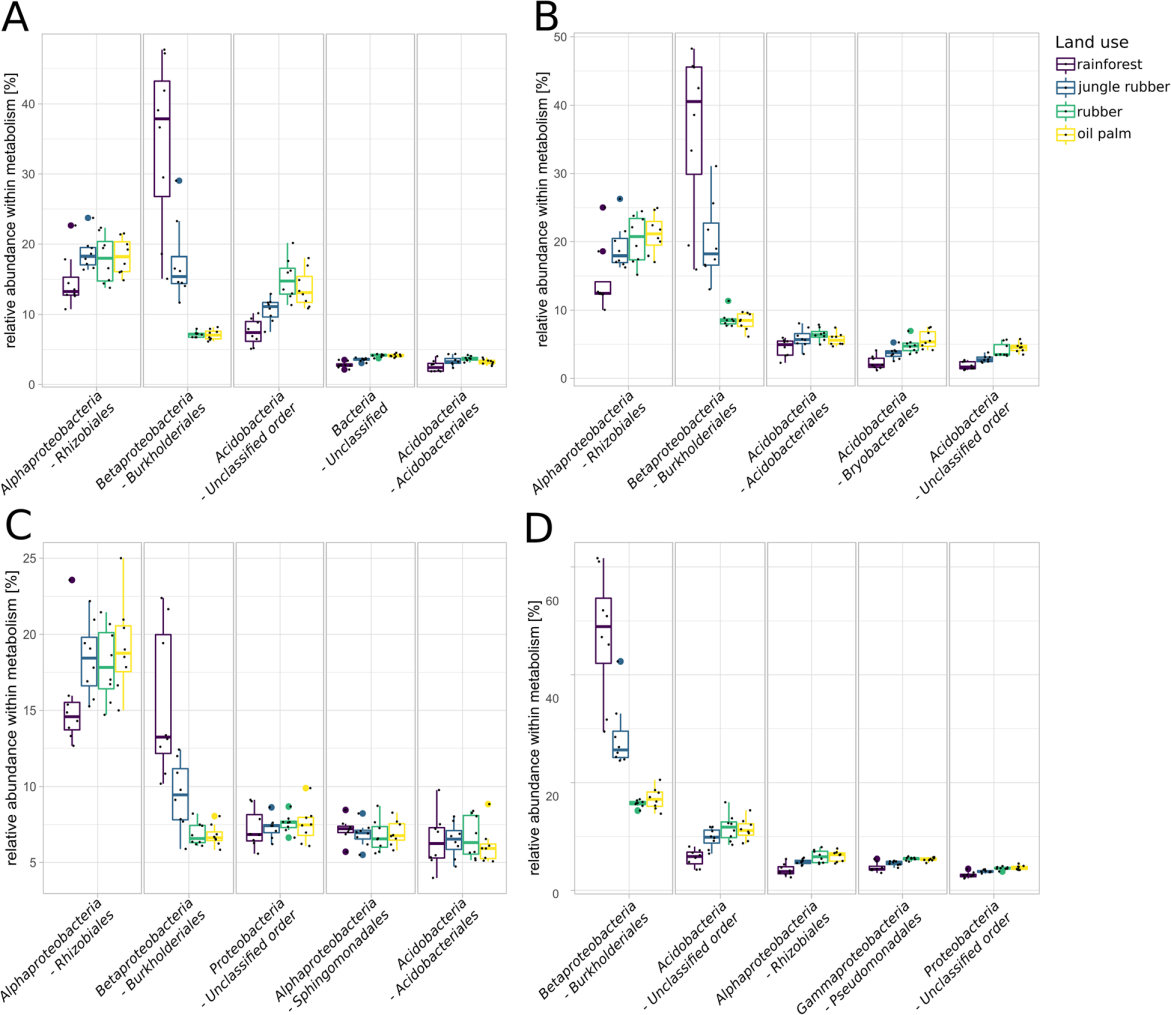
Relative abundances of the five most abundant taxonomic orders within bacterial chemotaxis (**a**), flagellar assembly (**b**), type IV secretion systems (**c**) and type VI secretion systems (**d**) KEGG level 3 categories in each land use system are displayed

To unravel the full scope of effects introduced by rainforest conversion on soil microbial community structure and functional profiles, it is necessary to identify which part of the present community is involved in which processes. In context of our previous analysis, we focussed on nitrogen metabolism (Fig. 8a), methane metabolism (Fig. 8b), bacterial chemotaxis (Fig. 9a), flagellar assembly (Fig. 9b), type IV secretion systems (Fig. 9c) and type VI secretion systems (Fig. 9d). Members of the *Rhizobiales* were prominent within nitrogen metabolism-related sequences in all samples with an average abundance of 12%. *Burkholderiales* were especially abundant in rainforest samples with 14.6% with a gradual abundance decrease from jungle rubber (8.4%) to rubber and oil palm samples (5.9%) (Fig. 8a). Both orders are known to be involved in various processes related to nitrogen metabolism and were previously identified as abundant groups in rainforest soils [40]. With unclassified *Acidobacteria*, and unclassified bacteria being high in abundance as well, observed patterns broadly reflected the general community structure as described before. However, redundant genes, which are involved in several pathways, are also present within broader functional categories. This likely explains the resemblance of the categories with the general community composition, including taxa usually not associated with typical nitrogen metabolism-related processes like *Acidobacteria*, which were still abundant in our analysed samples. Consequently, we identified taxa which are connected to previously analysed functional marker genes that showed significant changes in abundance due to rainforest conversion (Additional file 13: Figure S11, and Additional file 14: Figure S12). The taxonomic profiles for the denitrification-related genes *nirS* and *nirK* were distinct. *Rhizobiales* were the dominant identified phylogenetic group in *nirK* sequences of all samples with decreasing abundance from rainforest to converted land use systems. The *nirS* gene sequences were mostly associated to unidentified bacterial taxa, with a vast increase towards managed land use systems.

Similar patterns were observed for the methane metabolism-related taxa (Fig. 8b). However, unclassified acidobacterial sequences were more abundant in methane metabolism-related than in nitrogen metabolism-related taxa and showed abundance increases from rainforest to converted land use systems of 8.9 to 14.3%.

Taxa related to bacterial chemotaxis (Fig. 9a) and flagellar assembly (Fig. 9b) showed similar patterns in which *Rhizobiales* and *Burkholderiales* were the most abundant taxa. Again, *Burkholderiales* showed high abundances in rainforest samples. Unclassified acidobacterial orders were among the most abundant groups with abundance increases in converted land use systems for flagellar assembly-related and chemotaxis-related genes. Dominance of *Rhizobiales* and *Burkholderiales* in rainforest samples was also detected in type IV secretion system-related taxa (Fig. 9c). A slightly different pattern was observed for type VI secretion systems-related taxa (Fig. 9d), with *Burkholderiales* showing highest mean abundances in all samples.

## Discussion

### Dominant *Proteobacteria* and *Acidobacteria* are mostly affected by rainforest conversion

Observed decreases in taxonomic biodiversity accompanying rainforest conversion to managed land use systems were reported previously for various types of organisms [10, 11, 41, 42]. Diversity of bacteria and archaea decreased in plantation soils compared to rainforest. Contrary to these results, previous studies based on 16S rRNA gene analysis of the same sampling sites observed a diversity increase for bacteria from rainforest to converted land use systems [15, 16]. A higher amount of unclassified reads in shotgun datasets compared to 16S rRNA gene datasets could be a reason for the different results. Other studies focussing on nutrient cycling in these systems concluded that nutrient loss by leaching processes is elevated in rubber and oil palm plantation soils and affects bacterial diversity negatively [18, 43]. Fungal diversity increased with land use intensity and showed similar patterns as in a previous study [9]. The major difference between our direct metagenome sequencing-based approach and the other studies on the same sampling sites is that these were derived from 16S rRNA gene amplicon-based analysis. Since our study is based on metagenomic shotgun sequencing, we avoided possible primer bias and additionally have a higher sequencing depth, which both could lead to deviations between results derived from different approaches. Another reason for differences could be the classification of reads and the chosen tool for taxonomic binning. It has been reported that deviations in taxonomic profiles originate from false classifications rather than from sequencing errors [44]. Large-scale classifications of metagenomic short-reads are still a challenge though. To overcome possible shortcomings of single tools [44] and provide a higher classification efficiency [45], we combined the two classification tools Kraken2 and Kaiju to obtain robust results. General trends were confirmed by extracted 16S rRNA gene sequence controls and were similar to previous amplicon-based 16S rRNA gene studies [15, 16]. However, the fraction of unclassified bacterial taxa was larger in the extracted 16S rRNA gene-based control. This is the most notable differences to metagenomic shotgun data and demonstrates the advantage of a higher sequencing depth and consideration of all sequences instead of extracted marker gene sequences only.

The soil microbial community composition changed in the converted land use systems compared to rainforest with similar trends as in previous studies targeting these sampling sites [9, 15, 16]. *Proteobacteria* were negatively and *Acidobacteria* positively affected by rainforest conversion. The sharp decrease in abundance of *Rhizobiales* and *Burkholderiales* in converted systems suggests that they play a key role in rainforest soils, as suggested in previous studies [15, 16]. *Streptomycetales* are known for production of bioactive secondary metabolites and are abundant in endophytic communities [46]. Considering the reduction of plant diversity in the converted systems, the decrease of *Streptomycetales* accompanying rainforest conversion corresponds to the reduction of host diversity in the converted systems. The reduction of this group indicates that the overall biodiversity changes are reflected in abundance decreases of bacterial groups known to be involved in interspecific relationships as endophytes [47] or root nodule-forming symbionts [35], including the previously mentioned *Rhizobiales* and *Burkholderiales*.

Archaeal abundances did not seem to be affected by rainforest conversion. *Haloferacales* and *Halobateriales* were among the most abundant taxa and are known for their involvement in assimilatory nitrate reduction [48, 49]. However, we did not detect a significant involvement of these taxa in these processes, although we recorded significant changes of *narG* gene abundance. Based on our used methods, we could not identify a crucial effect of rainforest conversion on archaea. Although we did not find hints for functional importance in these systems, we cannot rule out the possibility of important archaeal activity due to the limitations of the DNA-based study design.

Our results showed that the fungal community composition in the studied land use systems was similar to an ITS-based analysis [9]. *Eurotiales* and *Hypocreales* are described as endophytes [50, 51], further supporting the assumption that the reduction of plant diversity also reduces endophyte occurrence. In addition to fungi, we analysed protists within the eukaryotes but the obtained trophic groups did not show significant differences in abundance in contrast to an 18S rRNA gene-based study [10]. This might be due to the differences between metagenomic shotgun sequencing and marker gene analysis [32]. In addition, protist sequences represented only a small portion (0.09%) in the entire metagenome dataset. Taking the number of unclassified sequences and the underrepresentation of protist genomes in the databases into account, the results for protists and other similar rare taxa should be taken with caution.

### Management-related soil characteristics shape soilborne communities

Multivariate analyses showed a similar topology for all analysed taxonomic groups, reflecting the proposed management and land use intensity gradient from rainforest over jungle rubber to rubber and oil palm. The correlation of abiotic factors LUI, pH, C:N ratio and Fe to the entire community as well as to bacteria is not surprising as the communities and correspondingly our dataset were dominated by bacterial sequences. These results are in accordance with other taxonomic marker gene analysis-based studies targeting bacteria, archaea [15, 16] and fungi [9] in the same study area. Management-related applications (e.g. fertilizer, liming and herbicides) are known to have an impact on C:N ratio and soil pH. These alterations in turn affect microbial communities [15, 52], which is also reflected by our results. Allen et al. [18] analysed the soil parameters in the used sampling sites and described decreases in organic matter stocks from rainforest to converted land use systems paralleled by a decreasing C:N ratio. Furthermore, nutrient availability for microbes is improved by fertilizer input in plantations, but is heavily dependent on ongoing fertilization, as these converted land use systems are more vulnerable to nutrient losses due to changes in soil texture and soil properties introduced by plantation of rubber and oil palm monocultures [18]. Therefore, our results regarding impact of rainforest conversion on community structure seem to be direct effects of the management practices in the converted land use systems. Consequently, the modification of the soil environment is a reason for the observed functional alterations (e.g. in nitrogen metabolism) caused by land use change and applied management practices.

### Functional profiles of energy and motility-related metabolisms are affected by rainforest conversion

In general, differential gene abundance analysis showed less changes in jungle rubber than in monoculture samples compared to rainforest. The gradually increase of changes reflects the land use gradient with stronger intensity in fertilized oil palm soils as proposed in previous studies [9, 15, 16].

Diversity of genes decreased with increasing land use intensity. Since most detected reads were of bacterial origin, it is not surprising that the functional diversity of the entire community shows similar results as bacterial diversity. For fungi, we detected a decrease in functional gene diversity, but an increase of taxonomic diversity, indicating an increase in less versatile species in monoculture soils or the presence of more different taxa equipped with similar gene sets.

We previously recorded that pH, carbon content, nitrogen content and C:N ratio played an important role for shaping the soil microbial communities. Our functional results support hypothesis (H2) that rainforest conversion affects metabolic reactions specific for nutrient cycling activity, including nitrogen metabolism and carbon fixation-related processes. As previously suggested, the change in nutrient availability affected soil microbial interaction and/or competition [16, 27]. Furthermore, it was hypothesized that different canopy structures in the analysed land use systems lead to higher light availability and higher ground temperatures in the converted systems, which in turn affect photosynthetic processes [15] and heat management of cells [16]. Detected higher abundance of photosynthesis related genes in oil palm further support this hypothesis.

The analysis of single functional marker genes in the metagenome dataset showed that denitrification genes *nirK* and *nirS* increased in abundance following rainforest conversion whereas nitrate reductase *narG* decreased. Allen et al. [18] described decreases in gross N mineralization, NH_4_^+^ immobilization and NH_4_^+^ pools in converted soils and generally higher N cycling rates in rainforest soils [18]. It was previously reported that *nirK* and *nirS* are positively correlated with increasing soil pH [53], which is in line with measurements conducted in our sampling sites [18] and multivariate analysis showing a significant influence of pH on microbial communities. Previous studies also described distinct distributions of the two different denitrification genes *nirK* and *nirS* among taxa, with *nirK* being associated to *Rhizobiales* [54]. Accordingly, most detected *nirK* sequences in our dataset originated from *Rhizobiales*. The higher gene abundances of denitrification-related genes in oil palm plantations correspond to previous observations in which higher N_2_O emissions, a by-product of denitrification, were detected in oil palm plantations [18].

We also observed higher nitrogen fixation potential in oil palm soils, which is surprising, considering previously reported nitrogen losses in these soils [18], although higher gene abundance is not necessarily accompanied by higher gene expression rates. Decreases in abundance of marker genes for flagellar assembly, chemotaxis, and type VI and IV secretion systems indicate that a reduction of motility and interaction in soil microbial communities accompanies rainforest conversion to intensively managed land use systems. It is challenging to identify the relationship of these aspects within a soil microbial community, since specific functions/processes can be associated with specific taxonomic groups. Our results indicate a shift from a dynamic community with higher motility, interaction and communication potential in rainforest soils to a community with less interaction potential in the managed plantations. This supports previous hypotheses regarding possible impacts of rainforest conversion towards community communication traits [16, 28]. The higher availability of nutrients in the fertilized plantation soils might lead to conditions in which competition for nutrients and the resulting high level of interspecific interaction and communication is not favoured or necessary and therefore reduced. Furthermore, specific taxonomic groups, involved in nutrient cycling, possibly show different abilities in terms of motility in the nutrient-supplemented planation soil compared to rainforest soil. This supports the theory of a less mobile community in planation soils but rather due to changes in community composition than to direct effects of rainforest conversion on functionality.

### Proteobacterial *Burkholderiales* are major players with respect to motility and nitrogen metabolism

We previously hypothesized that communities in rainforest soils are more dynamic and motile because of higher abundance of genes encoding flagellar assembly and chemotaxis. It is not surprising that a considerable number of reads belonging to these categories are assigned to abundant members of the community and we detected highest abundance on average for *Rhizobiales* in all samples. However, it is quite striking that the majority of flagellar assembly-related sequences in rainforest samples were assigned to members of *Burkholderiales* alone (Fig. 9). The same trend occurred for chemotaxis-related sequences. The degree of involvement in flagellar assembly leads to the suggestion that *Rhizobiales* and *Burkholderiales* are in general more capable of being motile than other groups in these soils. Additionally, significantly abundant genes connected to type IV and VI secretion systems were mostly derived from *Burkholderiales.* It was shown that members of the *Burkholderiales* are involved in co-migration with fungi in soil involving type IV pili [55]. The type VI secretion systems are involved in prokaryotic eukaryotic interactions [55–57]. Therefore, *Burkholderiales* and *Rhizobiales* possibly play an important functional role in soil microbial communities in rainforest due to their involvement in nutrient cycling. Their decrease in abundance in the converted land use systems was accompanied by various changes of the functional potential of the entire microbial soil community, which is affected by management-induced altered soil characteristics.

## Conclusion

We could show that rainforest conversion drastically affects structure and functional potential of soil microbial communities, which were dominated by bacteria. Taxonomic as well as functional diversity decreased for bacteria and archaea, whereas fungal biodiversity increased and functional diversity decreased, partly confirming hypothesis H1. Furthermore, functional profiles of the soil communities shifted along the land use gradient. Denitrification and nitrogen fixation potential increased with higher land use intensity, which is connected to abundant community members of *Burkholderiales* and *Rhizobiales*. Furthermore, we could show that pH and C:N ratio are drivers for shaping microbial community structure, which connects previously shown positive correlations of pH with land use intensity and denitrification potential. This is further supported by previous studies reporting an increase of N_2_O effluxes with rainforest conversion to oil palm plantations [18].

We detected a decrease in motility- and interaction-related functions from rainforest to converted land use systems indicating not only a shift in nutrient cycling activity but also in community dynamics confirming our second hypothesis (H2). Fertilizer application and higher short-term availability of nutrients in intensively managed plantations lead to an environment in which interspecific interactions apparently are less favoured compared to rainforest soil. Microbial communities underwent a shift in composition in which *Rhizobiales*, *Burkholderiales* and other members of the *Proteobacteria* decreased from rainforest to plantations, whereas *Acidobacteria* and *Actinobacteria* increased. Additionally, the largest fraction of sequences within analysed motility processes in rainforest belonged to members of the *Burkholderiales* and *Rhizobiales*, connecting decreases in abundance with a decrease in functions related to these processes (H3).

We could show connections between agricultural management and microbial community structure and functional potential in soil. Furthermore, this study provides a basis for further analysis on functional responses of soilborne microbial communities to rainforest conversion, which need ongoing attention due to the global impacts of large-scale land use changes in the tropics.

## Methods

### Study design and sampling site description

Sampling was conducted in two landscapes around the Harapan Rainforest Concession and the Bukit Duabelas National Park in midwest Sumatra (Indonesia) within the framework of the “Collaborative Research Centre 990: Ecological and Socioeconomic Functions of Tropical Lowland Rainforest Transformation Systems” (EFForTS). The two landscapes differ in soil texture, with clay acrisol soils in Bukit and loam acrisol soils in the Harapan landscape. Both landscapes harbour secondary lowland rainforest as reference site and three different converted land use systems: jungle rubber comprising planted rubber trees in secondary rainforest, rubber plantations (*Hevea brasiliensis* monocultures) and oil palm plantations (*Elaeis guineensis* monocultures). Rainforest was used as reference with low anthropogenic influence, while the three converted land use systems represent different land use intensities resulting in a land use intensity gradient (rainforest < jungle rubber < rubber < oil palm). Oil palm monoculture plantations were fertilized with 300 kg to 550 kg NPK fertilizer ha^− 1^ year^− 1^ [18]. Additionally, liming was performed in rubber and oil palm plantations with an average of 200 kg dolomite ha^− 1^ year^− 1^ and chemical and manual weeding was done by using Gramoxone and Roundup with an average of 2 to 5 L herbicide ha^− 1^ year^− 1^. Further information about the sampling sites and management of these sites is described by Allen et al. [18] and Brinkmann et al. [9]. Soil sampling, preparation (root removal), transport and storage was carried out as described in detail by Schneider et al. [15] and Berkelmann et al. [16]. Each of the four analysed land use systems (rainforest, jungle rubber, rubber plantation and oil palm plantation) consisted of four core plots per landscape, including three subplots (five by five meters) per core plot, resulting in 96 subplot samples and 32 core plots in total (Fig. 1). Abiotic data of the sampling sites were obtained from Allen et al. [18], Brinkmann et al. [9] (Additional file 15: Table S3).

### Sample preparation, extraction of bulk soil DNA and sequencing

For direct metagenome sequencing and prior to DNA extraction, soil samples were pooled in equal amounts at core plot level, resulting in 32 soil samples in total. DNA extraction of soil samples was performed with the MoBio Powersoil DNA extraction kit (MO BIO Laboratories Inc. Carlsbad, USA) as recommended by the manufacturer. The shotgun metagenomic sequencing of all DNA samples were performed on an Illumina HiSeq 4000 system with Nextera DNA Library Prep kits and paired-end reads of 2 × 150 bp as recommended by the manufacturer (Illumina, San Diego, USA).

### Taxonomic and functional assignment of paired-end reads

Raw sequences were quality-filtered with fastp (version 0.19.4) with a phredscore threshold of 20, overlapping base pair correction, sliding windows of 4 bp and a minimum length of 50 bp [58]. It has been shown that the combination of different sequence classifiers leads to more robust taxonomic assignments [44]. Therefore, we used Kraken2 and Kaiju in combination. Taxonomy assignments of short reads were performed by Kraken2 (v2.0.8-beta) [57] against the BLAST nt database (as of 2019-06-08). Afterwards unclassified reads were assigned with Kaiju (version 1.7.1) [59] against the BLAST nr database (as of 2019-06-08). Outputs were merged and taxonomy strings added by “addTaxonNames” by Kaiju (Additional file 16: Table S4). Protist groups were extracted from the normalized taxonomy table with “amp_subset_taxa” from the ampvis2 R package [60] according to taxonomy strings described by Schulz et al. [10]. Visualization of trophic groups was performed with ggplot2 [61]. A rRNA gene sequence-based control analysis was performed by extracting rRNA gene sequences from quality-filtered and merged reads employing sortmerna 2.1 with all by default available databases and settings [62]. Extracted rRNA gene sequences were classified as described above.

Assignment of read functions was done with previously quality-filtered reads (Additional file 17: Table S5). Functional classification was carried out by employing Kaijux [59] with default settings against the KEGG database [63] (as of 2018-10-01).

### Assigning the taxonomic background of identified functional genes

To assign the taxonomic affiliation of each sequence, taxonomic and functional assignments were further merged by combining the taxonomic classification with the obtained KEGG identifier for each sequence of the before-mentioned taxonomic read assignment and functional analysis, resulting in a per read taxonomy and function. This table was then filtered according to the targeted pathway, (i.e. nitrogen metabolism). All extracted hits were then normalized and displayed as relative abundances for the respective land use system with ggplot2.

### Analysis of selected functional metabolisms and respective marker genes

In order to analyse microbial functionality regarding rainforest conversion, we selected metabolisms and respective genes related to agricultural management induced changes [64]. Groups and genes of the KEGG database were used (KEGG level 3 for functional groups). We analysed functional pathways harbouring genes involved in nutrient cycling, including nitrogen metabolism, methane metabolism, carbon fixation and sulphur metabolism. For more detailed analyses, we picked marker genes and divided them into two categories regarding energy metabolism or motility. Genes selected for nitrogen metabolism were *amoA* (ammonia monooxygenase A; K10944) [65], *nifH* (nitrogenase protein; K02588) [40], *nosZ* (nitrous-oxide reductase; KK00376) [66], *nirK* and *nirS* (both encoding a nitrite reductase; K00368 and K15864) [40] and *narG* (nitrate reductase alpha subunit; K00370) [66]. For methane related processes we used *pmoA/amoA* (methane/ammonia monooxygenase subunit A; K10944) [64], *mxaF* (methanol dehydrogenase; K14028) [67], *mmoY* (methane monooxygenase component A beta chain; K16158) and *mmoX* (methane monooxygenase component A alpha chain; K16157) [68] and *mcrA* (5-methylcytosine-specific restriction enzyme A; K07451) [69].

To investigate effects on motility and interactions we analysed functional groups comprising flagellar assembly [70, 71] and chemotaxis [72], quorum sensing [73], and type IV and VI secretion systems [74, 75]. For flagellar assembly, we selected genes encoding flagellar motor switch proteins *fliN* (K02417), *fliM* (K02416) and *fliG* (K02410), the flagellar hook length protein *fliK* (K02414) [70] and a flagellar assembly protein *fliH* (K02411) [71]. Chemotaxis was covered by the sensor kinase gene *cheA* (K03407) [76], CheA response regulator *cheY* (KK03413) and scaffolding protein *cheW* (K03408) [77], CheY-phosphatase *cheX* (K03409) [78], and *cheR* (K00575), encoding a protein methyltransferase [79]. For quorum sensing we selected genes encoding cytoplasmic autoinducer receptors *luxR* (K10913), sensor kinases *luxQ* (K10909), *luxN* (K15850) and *luxS* (K07173) [80, 81]. Type IV secretion system related genes were represented by *virD4* (K03205) [82] and *virB4* (K03199) [83] and type VI secretion system related genes by *vgrG* (K11904) and *hcp* (K11903 [75].

### Statistical analyses

Diversity analysis, plotting of barplots and heatmaps were done with R [84] and RStudio [85] by using the packages ampvis2 [60], vegan [86], dplyr [61], stringr and ggplot2 [61]. Shannon diversity index calculation for taxonomic diversity was performed with previously rarefied data using ampvis2 with amp_alphadiv. Functional diversity was calculated by summing all identical genes of the entire community or by summing all identical genes in the respective domain before calculating the Shannon index with ampvis2. Statistical analysis of calculated Shannon diversity results was performed with the vegan package (version 2.5–5). First, the Shapiro test was used to determine normal distribution of the data with base R and shapiro.test. All obtained values were non-normally distributed and therefore further analysed with base R by using the Kruskal-Wallis test (Kruskal.test) with subsequent pairwise Wilcoxon test (pairwise.wilcox.test).

Differential abundance analysis and count normalization of taxonomic or functional data were performed by using the DESeq2 package [87]. Default settings with the Benjamini and Hochberg correction were used after removing singletons. Normalized counts were extracted as described in the package manual and used for data visualizations. Differential abundance analysis was done for the entire dataset by the main DESeq function. Differential abundance results were obtained by the “contrast” function, in which log2fold changes of rainforest compared to each converted land use were extracted in a pairwise fashion (results_dataframe <− results(dds, contrast = c(“condition”, “reference”, “treatment”)), resulting in positive log2fold changes when gene abundances increased from reference to the respective land use and negative values when abundances decreased. The threshold for significant differences was set to *p* < 0.05. Generated tables per used condition were merged by using the dplyr package and visualized by ggplot2. Analysis was performed in a same manner for taxonomic and functional classifications. Differences in abundances between rainforest and each land use were tested at order level. Functional profiles were tested at metabolism level (level 3 of the KEGG hierarchy) and gene level.

Obtained taxonomy profiles of extracted rRNA sequences were analysed for similarity to shotgun data by calculating Bray Curtis dissimilarity matrices with the “adonis” function of the vegan package in R and subsequent tests by using pairwise PERMANOVA tests with “pairwise.perm.manova” of the RVAideMemoire package [88] in R.

Ordination analysis was performed with the ampvis2 package [60]. Raw data was rarefied for each tested taxonomic group or the entire community by ampvis2 (amp_subset_samples) before a Bray Curtis dissimilarity matrix was calculated and visualized by Nonmetric multidimensional scaling (NMDS). Data for soil characteristics of the sampling sites were obtained from Allen et al. [18] (Additional file 11: Table S3). In addition to abiotic measurements, we also included the Land Use Index (LUI) as described by Brinkmann et al. [9] (Additional file 15: Table S3). An environmental fit was calculated with the “amp_ordinate” function in ampvis2 with an envfit significance level of p < 0.05.

## Supplementary information

**Additional file 1: Table S1.** Sequence counts of all samples before and after quality-filtering with fastp.

**Additional file 2: Figure S1.** Relative abundances of each domain for each analysed land use system in the respective landscape.

**Additional file 3: Figure S2.** Distribution of taxonomic differences compared to rainforest at order level. Differences are displayed as log2fold (treatment vs control) changes with p.adj < 0.05 for detected orders in all land use systems. Bukit jungle rubber is not depicted as no significant changes of taxonomic orders were detected.

**Additional file 4: Figure S3.** Composition of different trophic protist groups. Trophic groups were assigned according to Schulz et al. [10]. Compositions are displayed for each land use in the respective landscape as relative abundances.

**Additional file 5: Figure S4.** Community composition at order level based on extracted rRNA sequences (A) and all obtained reads by shotgun sequencing (E). orders with relative abundances below 1% were clustered as “rare taxa”. The five most abundant orders based on average relative abundance of extracted 16S rRNA gene sequences in all treatments are shown for bacteria (B), archaea (C) and fungi (D) as well as for all obtained reads by shotgun sequencing (F-H).

**Additional file 6: Table S2.** Environmental fit results for the entire community, bacteria, archaea and fungi.

**Additional file 7: Figure S5.** Distribution of gene changes in each converted land use system compared to rainforest. Detected changes are displayed as log2fold changes (treatment vs control; p.adj < 0.05).

**Additional file 8: Figure S6.** Principal Component Analysis for detected genes of all analysed land use systems and rainforest samples in the respective landscape. PCA analysis is based on transformed counts by using the regularized log function of DESeq2.

**Additional file 9: Figure S7.** Functional profile of selected metabolisms based on KEGG categories (level 3) displayed as log2fold changes in separated landscapes. Negative log2fold changes indicate higher abundances in rainforest samples, whereas positive log2fold changes indicate higher abundance in the corresponding converted land use systems. Values with p.adj < 0.05 are marked with *.

**Additional file 10: Figure S8.** Log2fold changes of selected marker genes of nitrogen and methane metabolism for each analysed land use system compared to rainforest for each separate landscape. Significant differences (p.adj. < 0.05) are marked with *. Negative log2fold changes indicate higher abundances in rainforest samples, whereas positive log2fold changes indicate higher abundance in the corresponding converted land use systems.

**Additional file 11: Figure S9.** Log2fold changes of selected marker genes motility related marker genes for each analysed land use system compared to rainforest in each landscape. Significant differences (p.adj. < 0.05) are marked with *. Negative log2fold changes indicate higher abundances in rainforest samples, whereas positive log2fold changes indicate higher abundance in the corresponding converted land use systems.

**Additional file 12: Figure S10.** Log2fold changes of *nod* genes in each land use compared to rainforest. Significant differences (p.adj < 0.05) are marked with *. Negative log2fold changes indicate higher abundances in rainforest samples, whereas positive log2fold changes indicate higher abundance in the corresponding converted land use systems.

**Additional file 13: Figure S11.** Relative abundances of the five most abundant detected taxonomic orders within all detected sequences for nitrogen related marker genes that showed significant differences between rainforest and converted land use systems. Displayed heatmaps show the five most abundant detected taxa for nitrite reductase gene *nirK*, nitrite reductase gene *nirS* and nitrate reductase alpha subunit gene *narG*.

**Additional file 14: Figure S12.** Relative abundances of the five most abundant detected taxonomic orders within all detected sequences for motility related marker genes that showed significant differences between rainforest and converted land use systems.

**Additional file 15: Table S3.** Used abiotic measurements for all samples.

**Additional file 16: Table S4.** Taxonomic counts of all analysed sequences. The first column shows ID and the last column the detected taxonomy.

**Additional file 17: Table S5.** Count matrix containing functional assignments for all samples. The first column shows the ID the last columns the function according to the KEGG database including the KO number and the full pathway description.

## Data Availability

All data generated or analysed during this study are included in this published article. Obtained sequences were deposited in the National Center for Biotechnology Information (NCBI) Sequence Read Archive (SRA) under accession number PRJNA562410.
