# Supplementary figures and images for: Unravelling the effects of tropical land use conversion on the soil microbiome
Source: Environ Microbiome. 2020 Feb 3;15:5. doi: 10.1186/s40793-020-0353-3 (PMC8067294; doi:10.1186/s40793-020-0353-3)

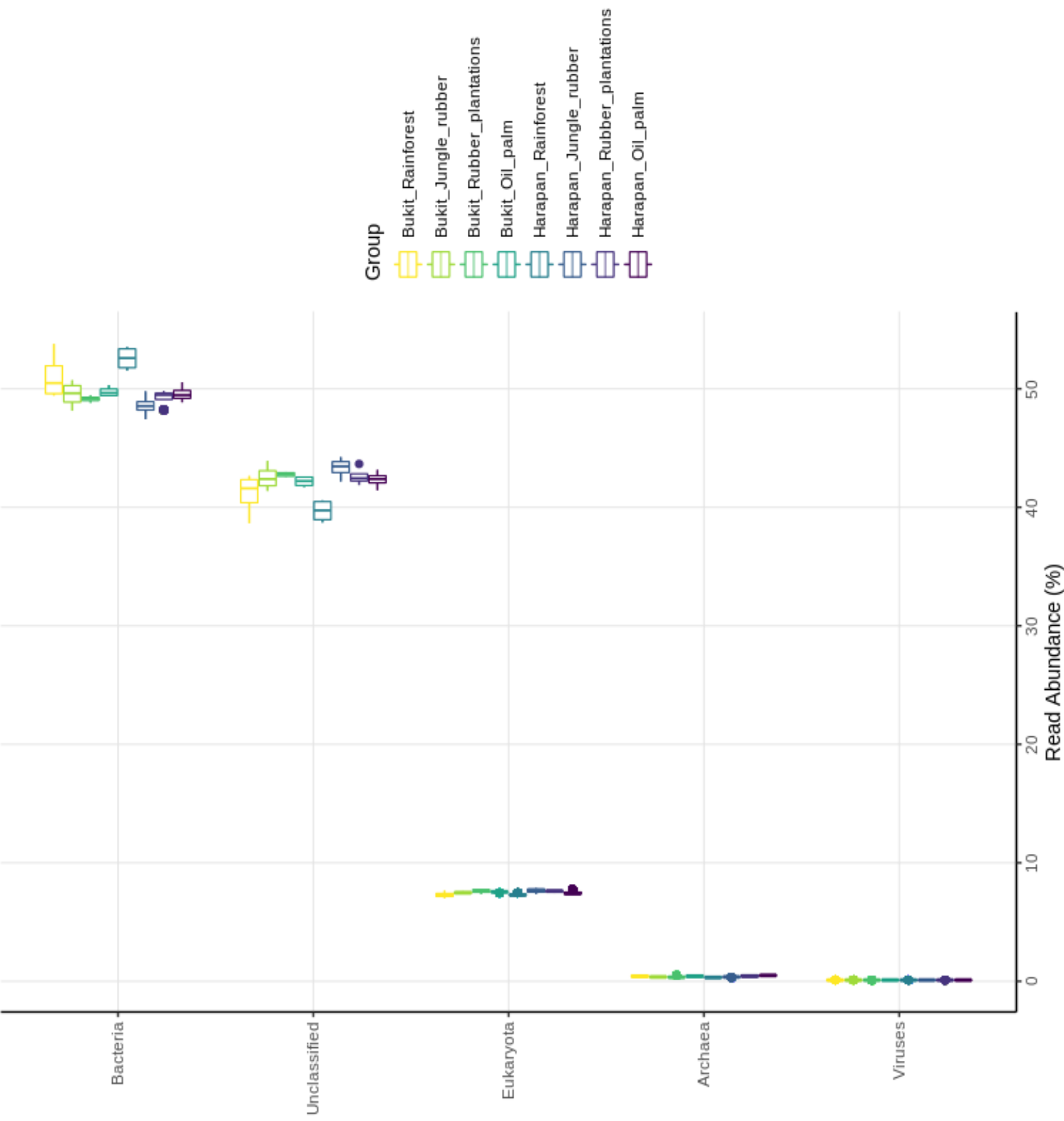

Supplement: Supplementary file 2 — Additional file 2: Figure S1. Relative abundances of each domain for each analysed land use system in the respective landscape. [file 40793_2020_353_MOESM2_ESM.pdf]

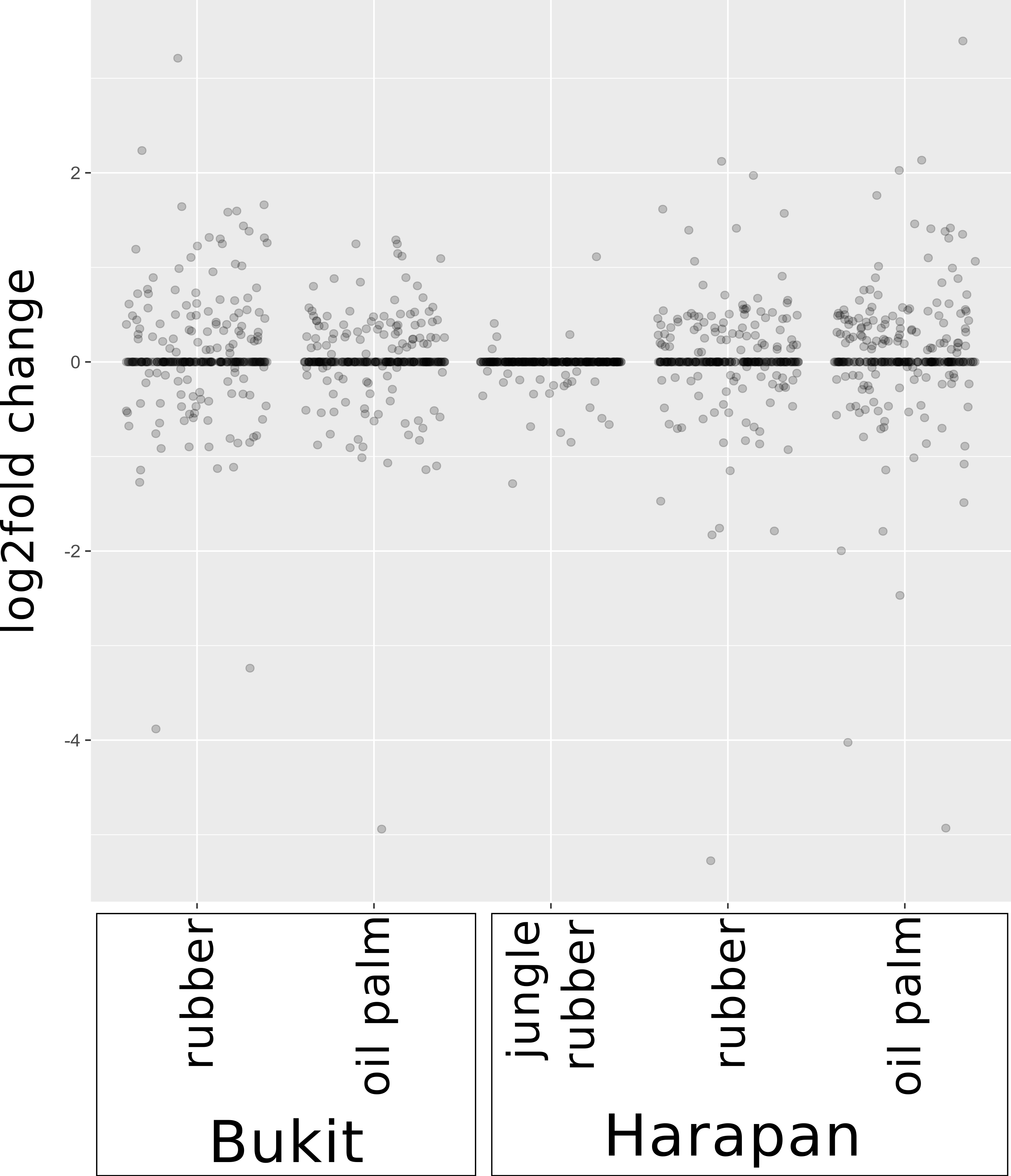

Supplement: Supplementary file 3 — Additional file 3: Figure S2. Distribution of taxonomic differences compared to rainforest at order level. Differences are displayed as log2fold (treatment vs control) changes with p.adj < 0.05 for detected orders in all land use systems. Bukit jungle rubber is not depicted as no significant changes of taxonomic orders were detected. [file 40793_2020_353_MOESM3_ESM.png]

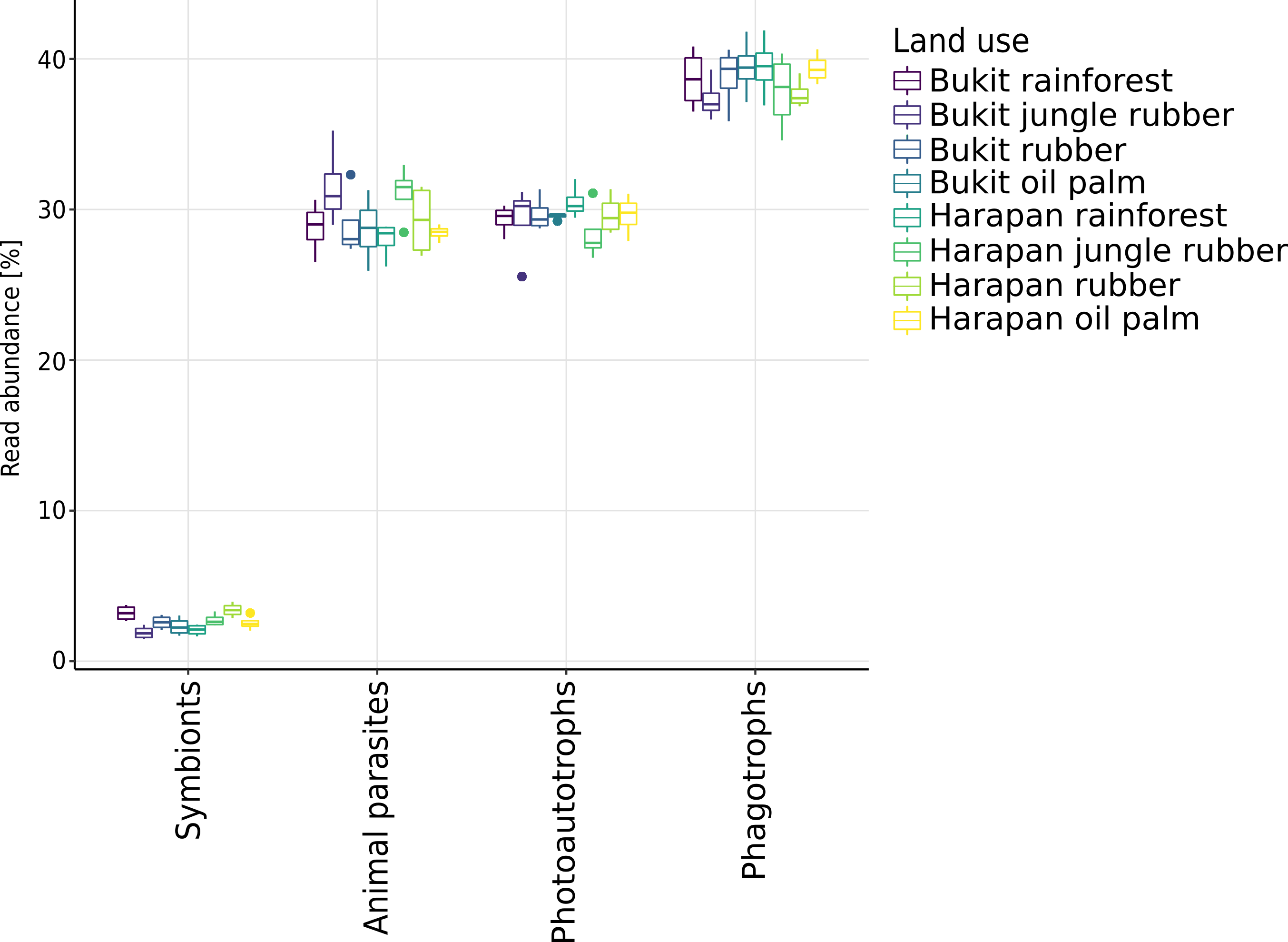

Supplement: Supplementary file 4 — Additional file 4: Figure S3. Composition of different trophic protist groups. Trophic groups were assigned according to Schulz et al. [10]. Compositions are displayed for each land use in the respective landscape as relative abundances. [file 40793_2020_353_MOESM4_ESM.png]

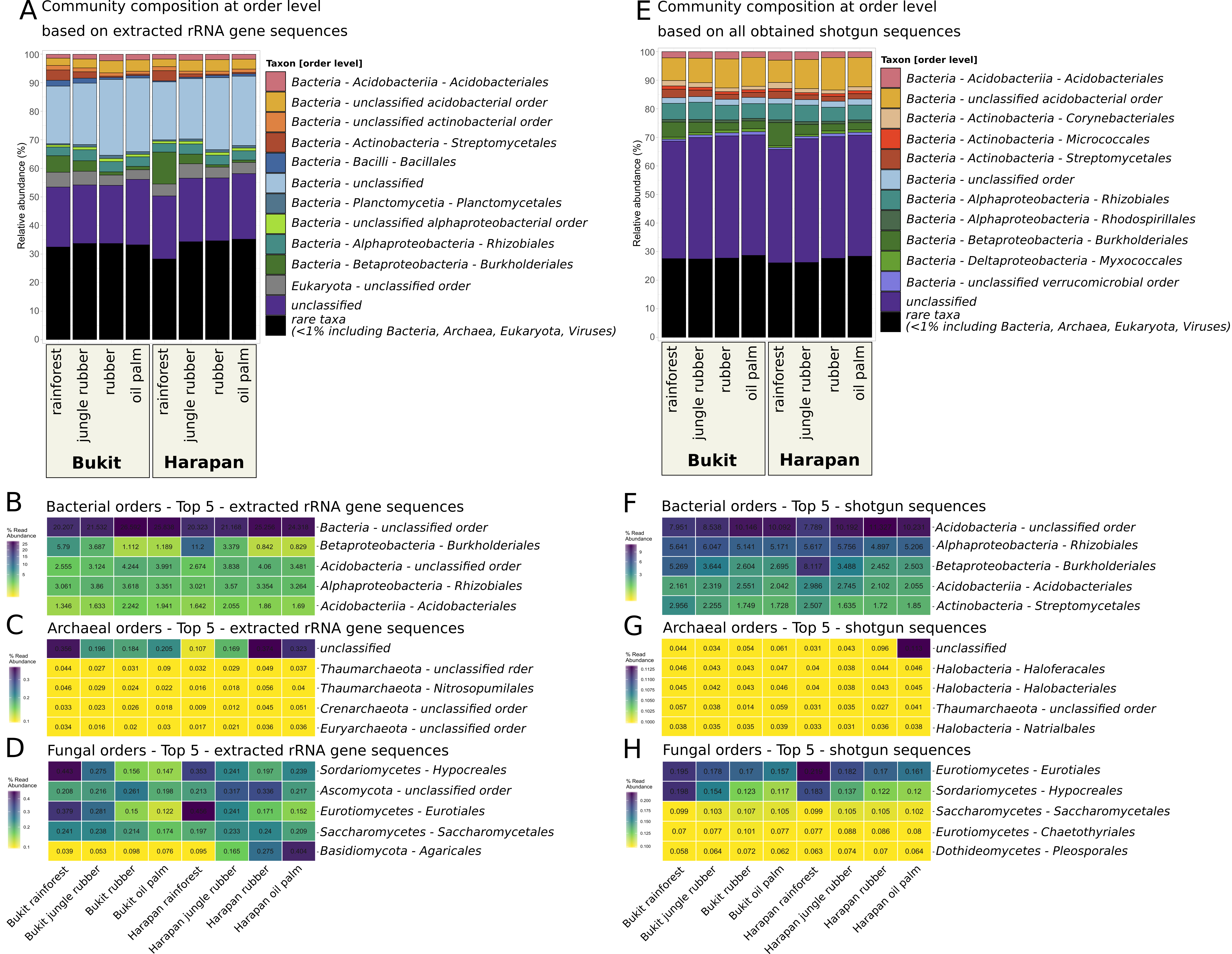

Supplement: Supplementary file 5 — Additional file 5: Figure S4. Community composition at order level based on extracted rRNA sequences (A) and all obtained reads by shotgun sequencing (E). orders with relative abundances below 1% were clustered as “rare taxa”. The five most abundant orders based on average relative abundance of extracted 16S rRNA gene sequences in all treatments are shown for bacteria (B), archaea (C) and fungi (D) as well as for all obtained reads by shotgun sequencing (F-H). [file 40793_2020_353_MOESM5_ESM.png]

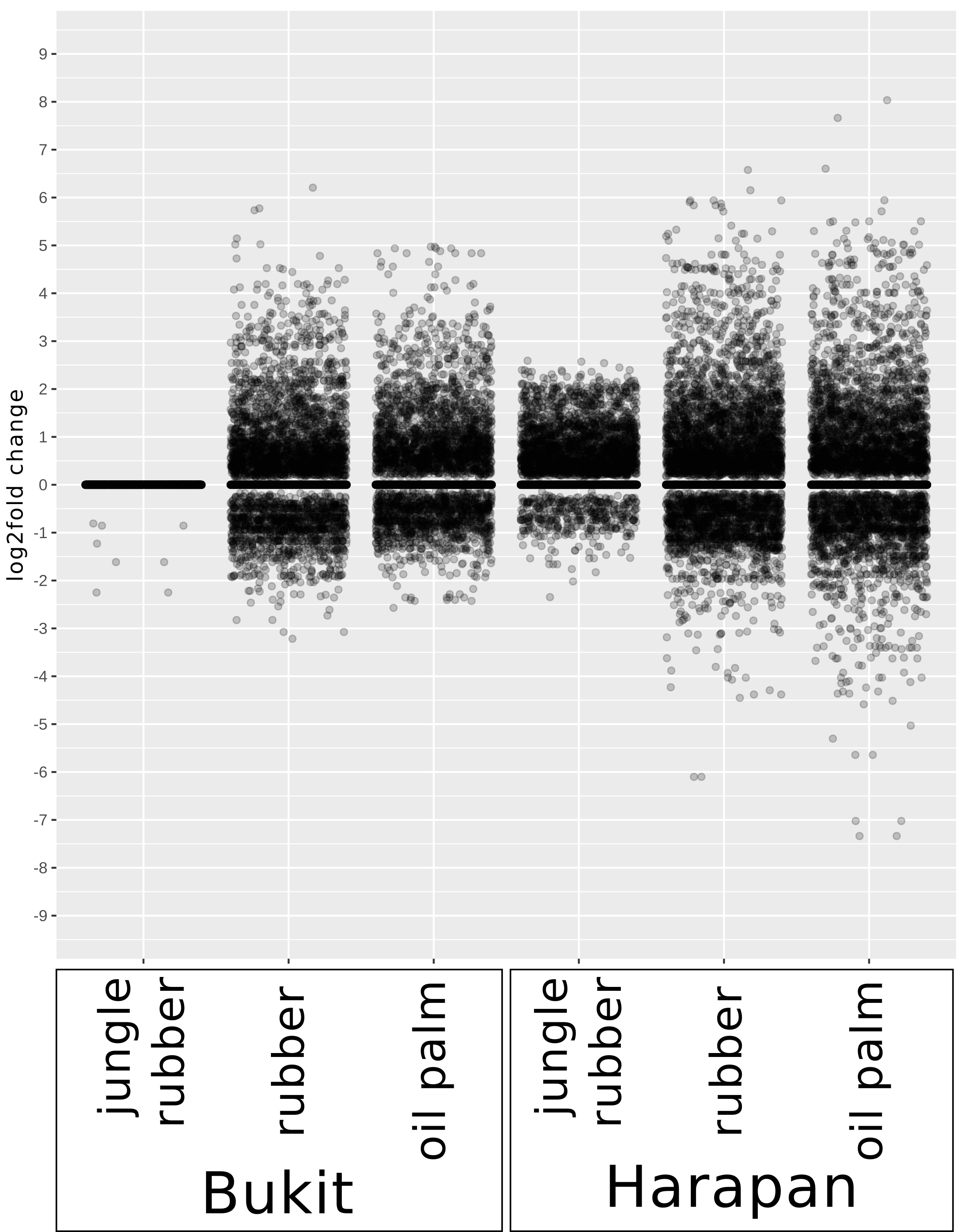

Supplement: Supplementary file 7 — Additional file 7: Figure S5. Distribution of gene changes in each converted land use system compared to rainforest. Detected changes are displayed as log2fold changes (treatment vs control; p.adj < 0.05). [file 40793_2020_353_MOESM7_ESM.png]

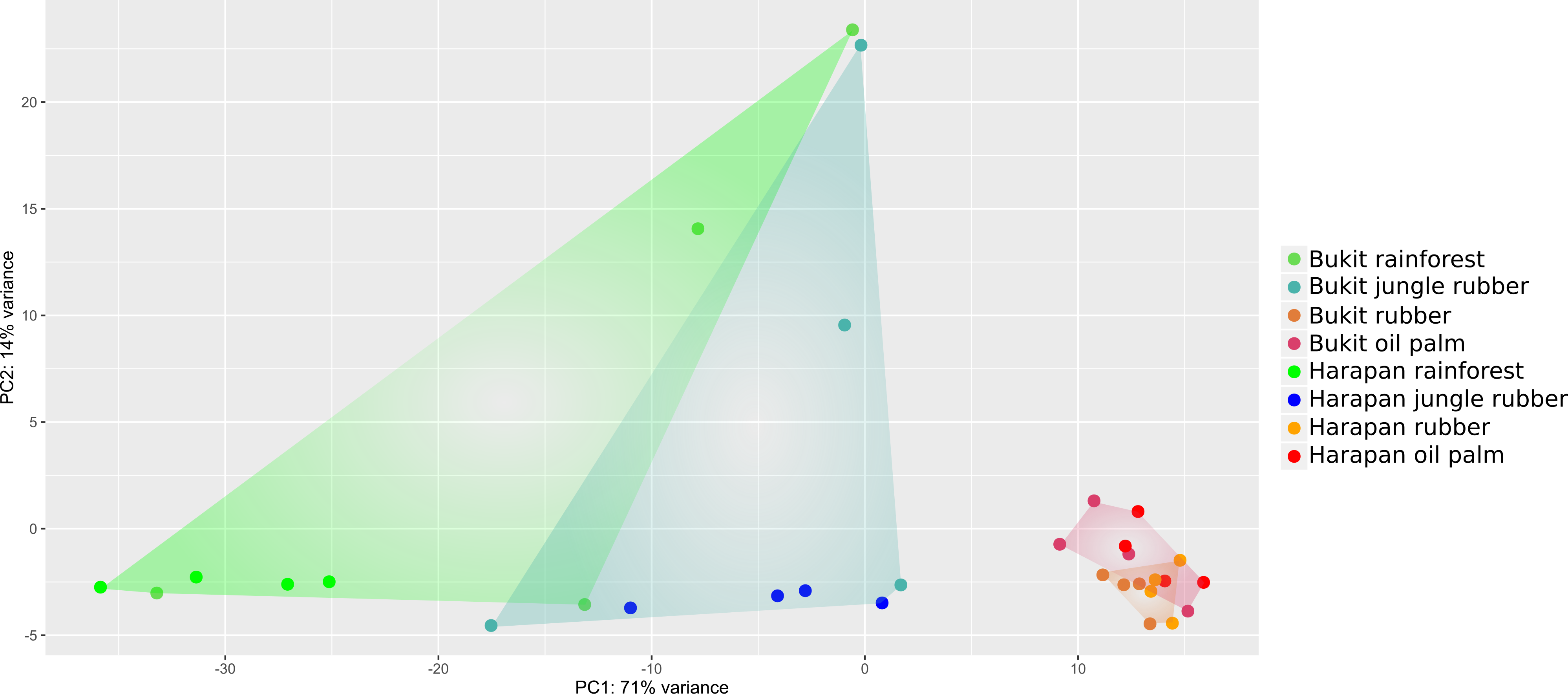

Supplement: Supplementary file 8 — Additional file 8: Figure S6. Principal Component Analysis for detected genes of all analysed land use systems and rainforest samples in the respective landscape. PCA analysis is based on transformed counts by using the regularized log function of DESeq2. [file 40793_2020_353_MOESM8_ESM.png]

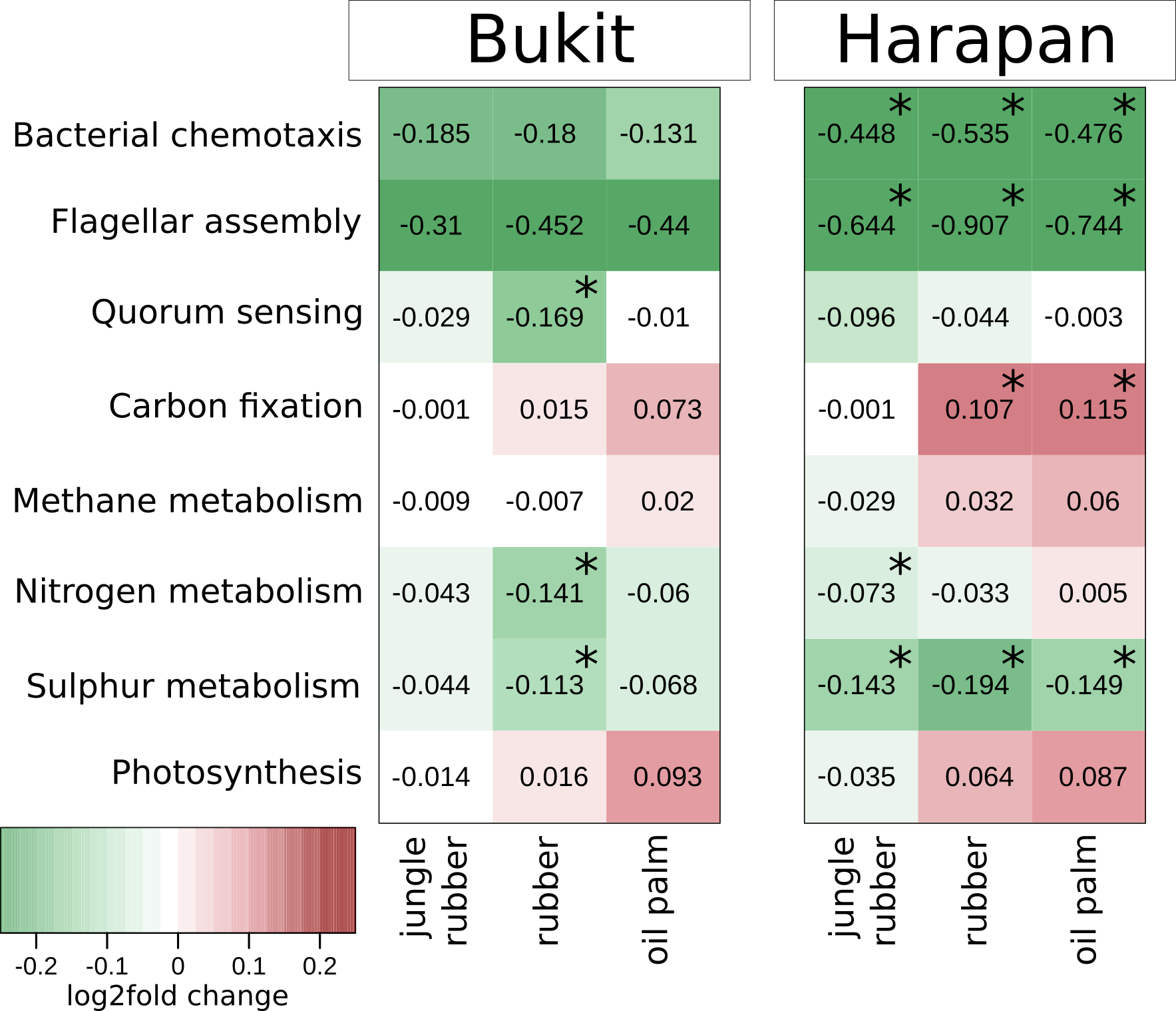

Supplement: Supplementary file 9 — Additional file 9: Figure S7. Functional profile of selected metabolisms based on KEGG categories (level 3) displayed as log2fold changes in separated landscapes. Negative log2fold changes indicate higher abundances in rainforest samples, whereas positive log2fold changes indicate higher abundance in the corresponding converted land use systems. Values with p.adj < 0.05 are marked with *. [file 40793_2020_353_MOESM9_ESM.png]

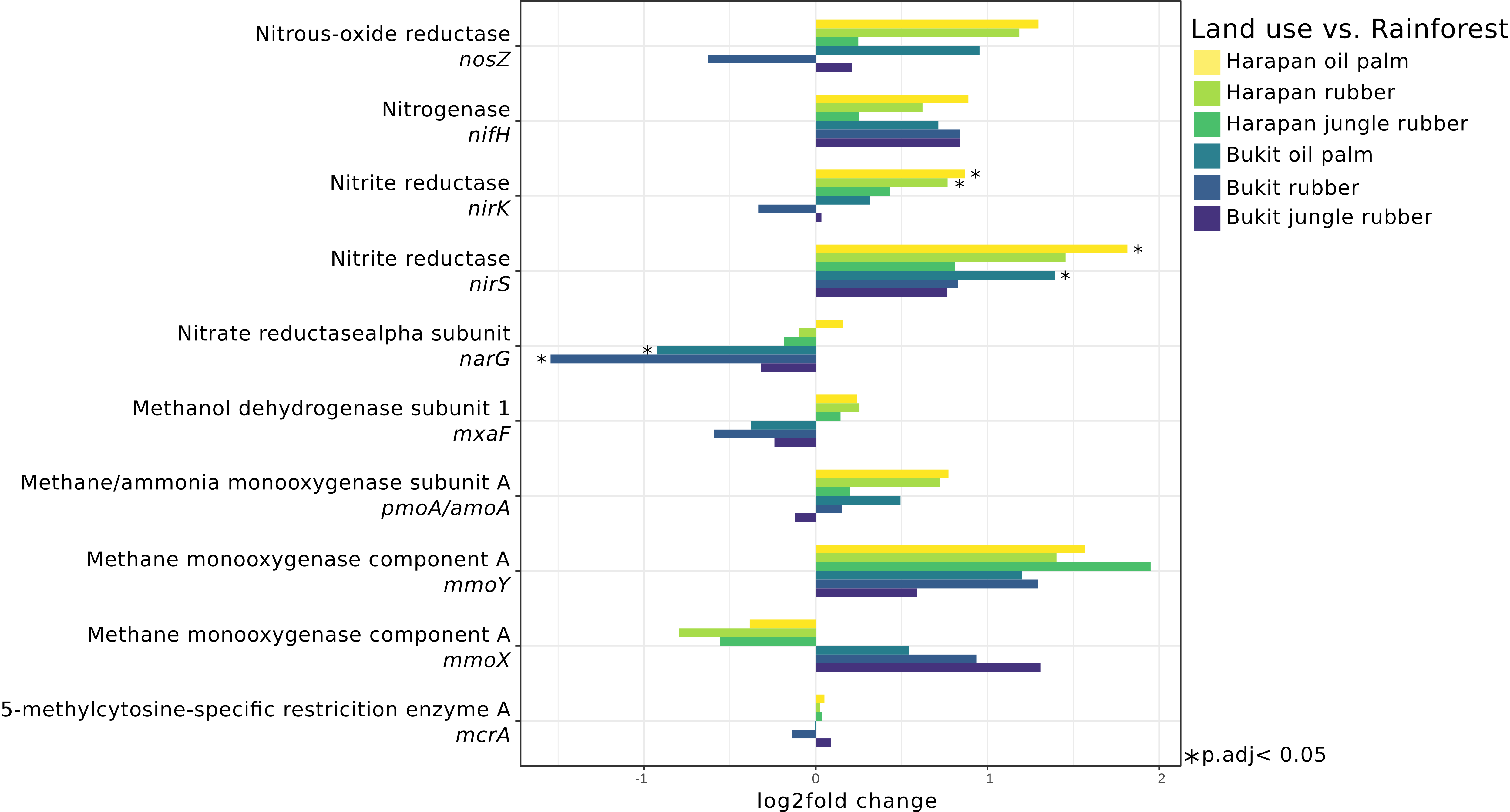

Supplement: Supplementary file 10 — Additional file 10: Figure S8. Log2fold changes of selected marker genes of nitrogen and methane metabolism for each analysed land use system compared to rainforest for each separate landscape. Significant differences (p.adj. < 0.05) are marked with *. Negative log2fold changes indicate higher abundances in rainforest samples, whereas positive log2fold changes indicate higher abundance in the corresponding converted land use systems. [file 40793_2020_353_MOESM10_ESM.png]

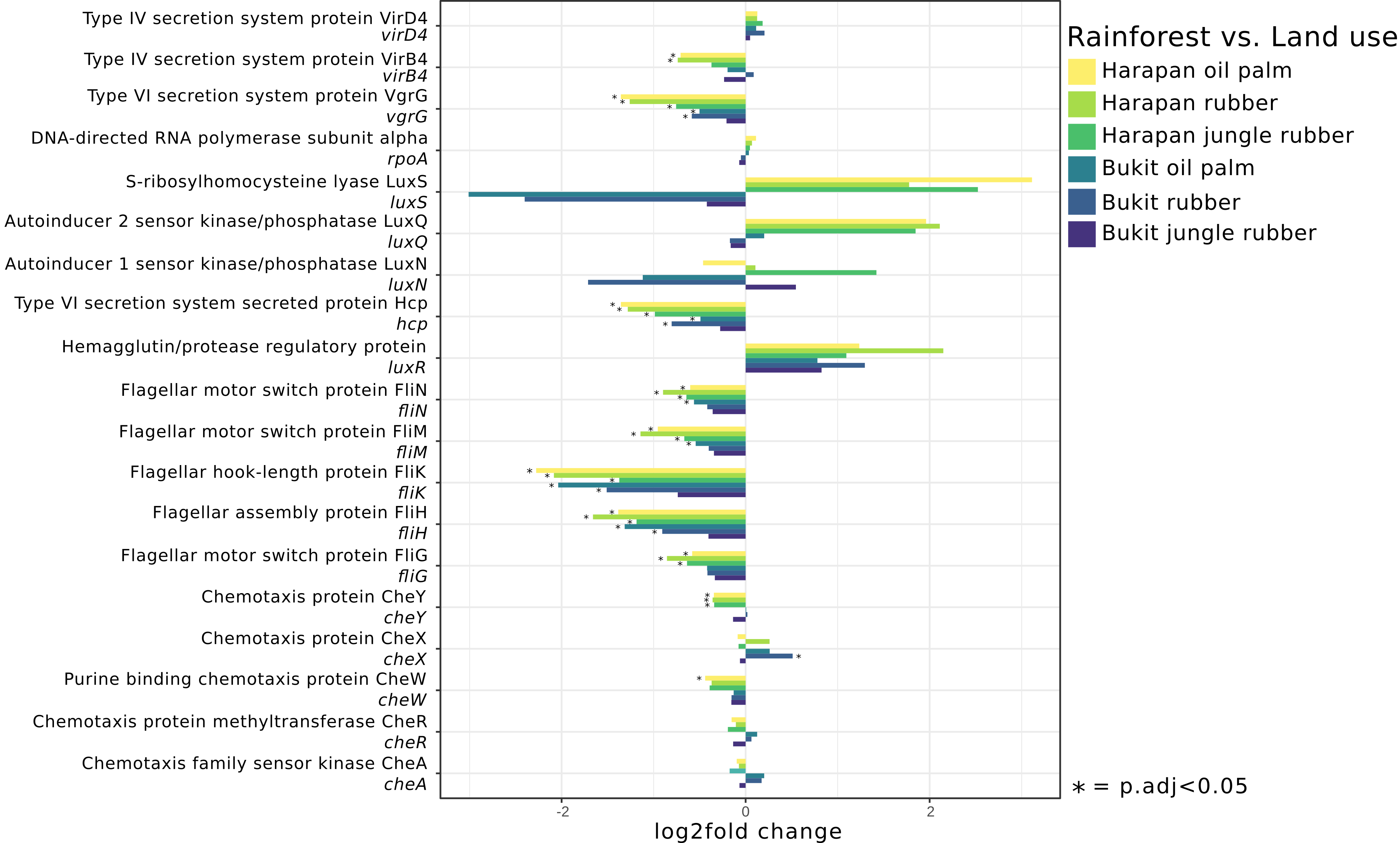

Supplement: Supplementary file 11 — Additional file 11: Figure S9. Log2fold changes of selected marker genes motility related marker genes for each analysed land use system compared to rainforest in each landscape. Significant differences (p.adj. < 0.05) are marked with *. Negative log2fold changes indicate higher abundances in rainforest samples, whereas positive log2fold changes indicate higher abundance in the corresponding converted land use systems. [file 40793_2020_353_MOESM11_ESM.png]

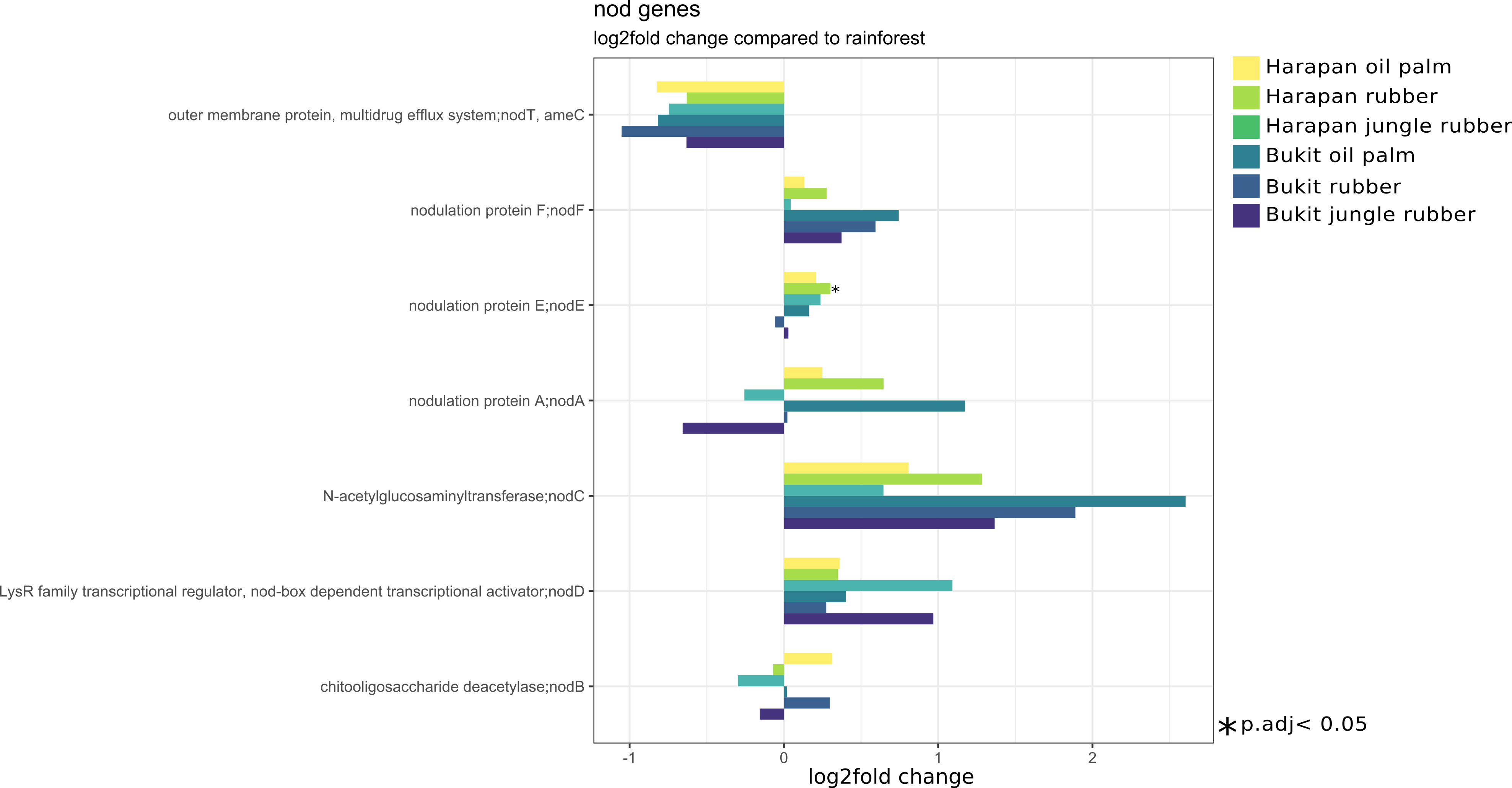

Supplement: Supplementary file 12 — Additional file 12: Figure S10. Log2fold changes of nod genes in each land use compared to rainforest. Significant differences (p.adj < 0.05) are marked with *. Negative log2fold changes indicate higher abundances in rainforest samples, whereas positive log2fold changes indicate higher abundance in the corresponding converted land use systems. [file 40793_2020_353_MOESM12_ESM.png]

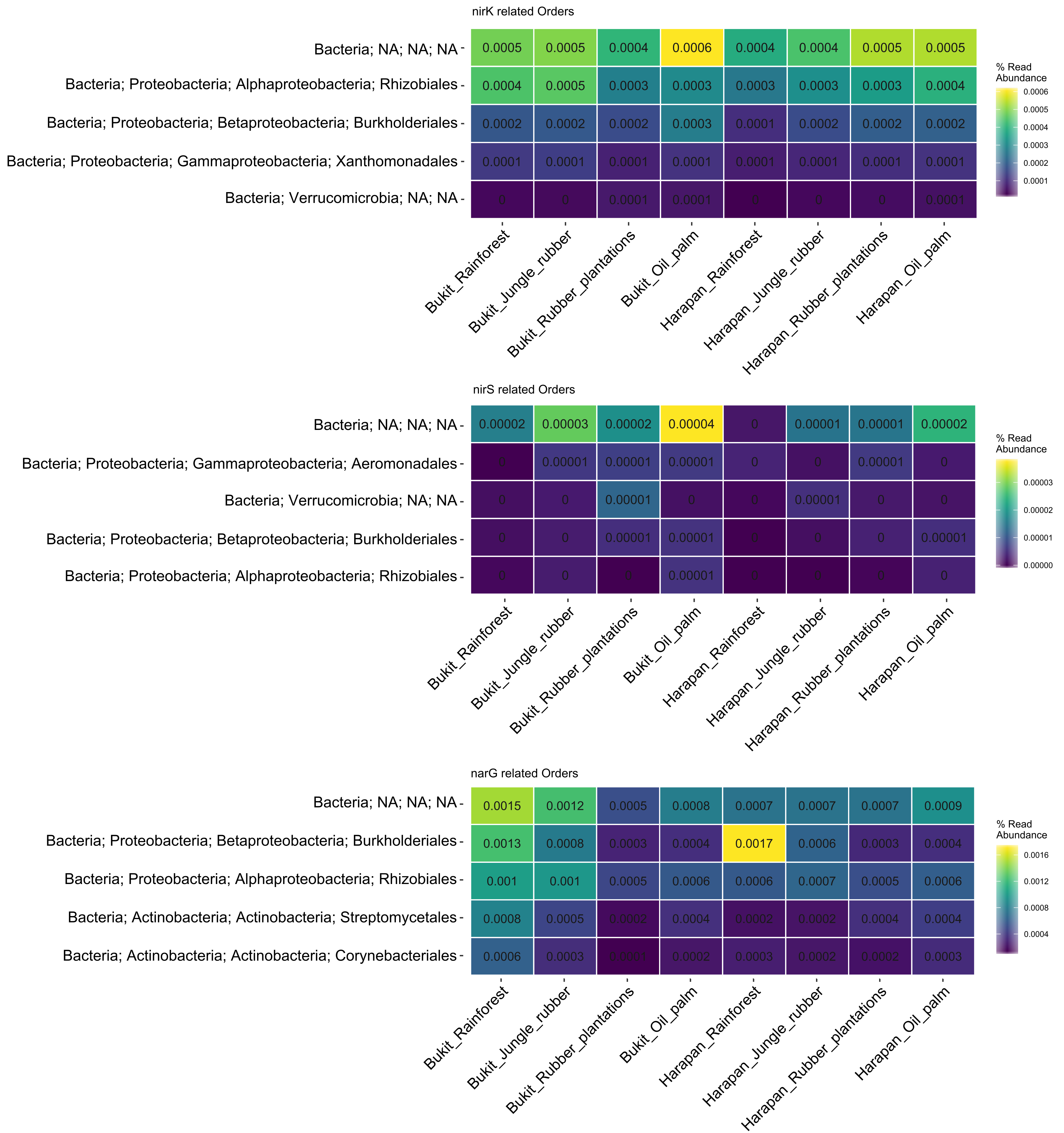

Supplement: Supplementary file 13 — Additional file 13: Figure S11. Relative abundances of the five most abundant detected taxonomic orders within all detected sequences for nitrogen related marker genes that showed significant differences between rainforest and converted land use systems. Displayed heatmaps show the five most abundant detected taxa for nitrite reductase gene nirK, nitrite reductase gene nirS and nitrate reductase alpha subunit gene narG. [file 40793_2020_353_MOESM13_ESM.png]

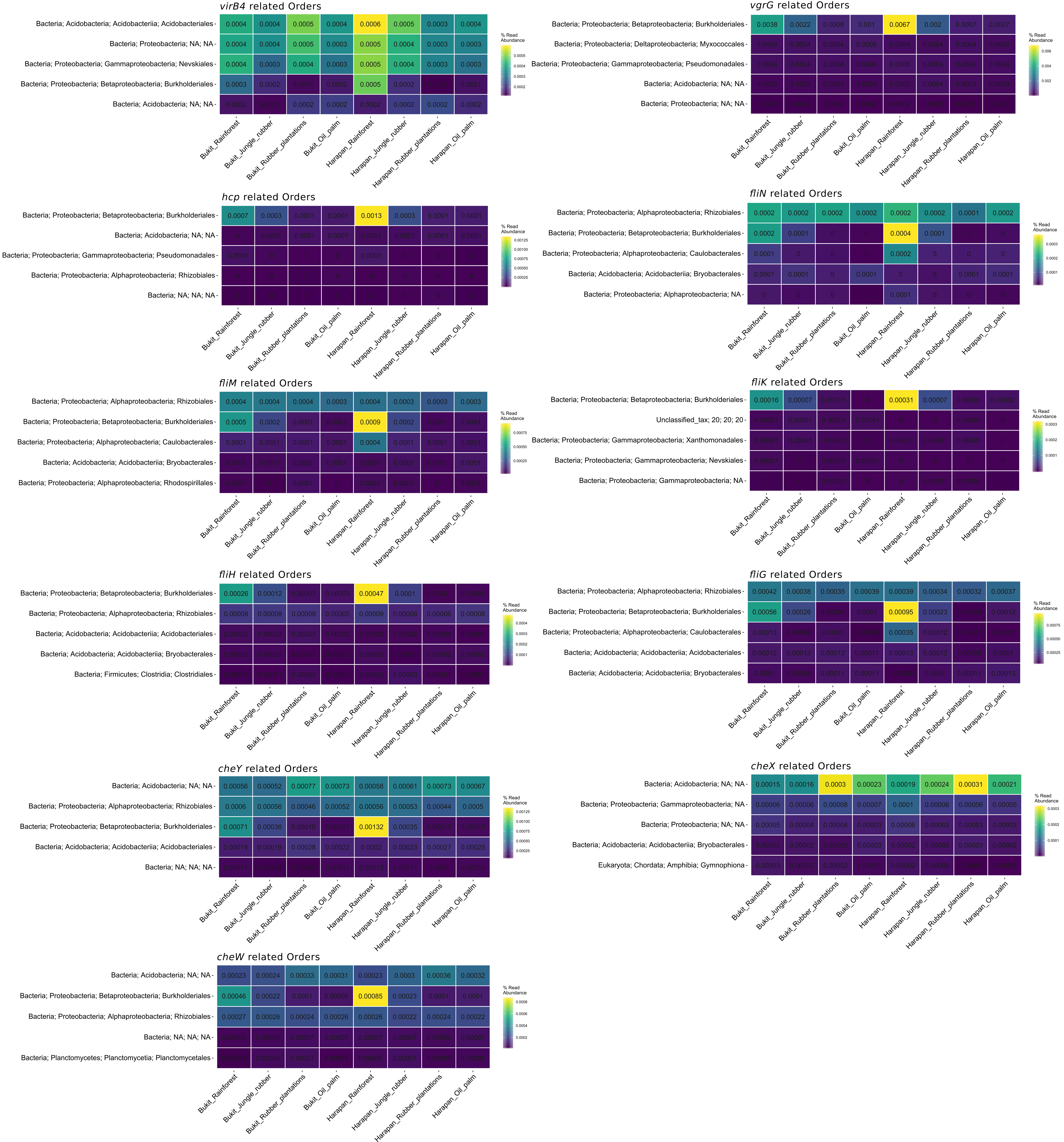

Supplement: Supplementary file 14 — Additional file 14: Figure S12. Relative abundances of the five most abundant detected taxonomic orders within all detected sequences for motility related marker genes that showed significant differences between rainforest and converted land use systems. [file 40793_2020_353_MOESM14_ESM.png]
